# TRIM65-BTG3-HIF-1α axis drives osteosarcoma progression *via* M2 macrophage polarization and angiogenesis

**DOI:** 10.1515/jtim-2026-0075

**Published:** 2026-10-23

**Authors:** Hao Wang, Rui Yang, Zhongxuan Wu, Mian Zhang, Longze Xiao, Yukang Que, Peng He, Qiming Ma, Tangbing Xu, Chi Zhang, Shenglin Xu, Yong Hu

**Affiliations:** Department of Orthopedics, The First Affiliated Hospital of Anhui Medical University, Hefei, Anhui Province, China; Department of Orthopedics, Anhui Public Health Clinical Center, Hefei, Anhui Province, China; Clinical College of Anhui Medical University, Anhui Medical University, Hefei, Anhui Province, China

**Keywords:** osteosarcoma, tumor-associated macrophages, B-cell translocation gene 3, tripartite motif containing 65, hypoxia-inducible factor 1α, angiogenesis

## Abstract

**Background and Objectives:**

Osteosarcoma (OS) is an aggressive bone cancer with a poor prognosis, especially in metastatic cases. The tumor microenvironment (TME), particularly tumor-associated macrophages (TAMs), plays a crucial role in its progression. This study aimed to investigate the role and mechanism of the tripartite motif-containing 65 (TRIM65), B-cell translocation gene 3 (BTG3), hypoxia-inducible factor 1α (HIF-1α; TRIM65-BTG3-HIF-1α) signaling axis in regulating TAMs and angiogenesis in OS.

**Methods:**

Bioinformatic analysis of The Cancer Genome Atlas (TCGA) and TARGET-OS datasets identified M2 macrophage-related genes. Clinical OS samples were analyzed *via* immunohistochemistry (IHC), immunofluorescence (IF), messenger ribonucleic acid (mRNA) sequencing, and data-independent acquisition (DIA) quantitative proteomics. *In vitro* functional assays (cell counting kit-8 [CCK-8], colony formation, transwell migration/invasion, tube formation) and *in vivo* subcutaneous xenograft models in nude mice were performed using OS cell lines (U-2 OS [U2OS], sarcoma osteogenics [SAOS], human OS (HOS), 143B) and the human fetal osteoblast (hFOB) normal osteoblast line. Macrophage polarization was induced from Tohoku Hospital pediatrics-1 (THP-1) cells. Molecular interactions were assessed by co-immunoprecipitation (Co-IP), ubiquitination assays, and luciferase reporter assays.

**Results:**

High infiltration of M2 macrophages correlated with poor patient prognosis. BTG3 was identified as a key M2-related gene exhibiting a "transcriptional upregulation with translational downregulation" pattern in OS. TRIM65, an E3 ubiquitin ligase upregulated in OS, directly interacted with and promoted the ubiquitin-mediated degradation of the BTG3 protein. Knockdown of TRIM65 inhibited OS cell proliferation, migration, invasion *in vitro*, and tumor growth *in vivo*. BTG3 acted as a negative regulator of HIF-1α transcriptional activity. Loss of BTG3 led to HIF-1α activation, which in turn promoted M2 polarization and enhanced migration of macrophages. Furthermore, conditioned medium from BTG3-deficient cells significantly promoted endothelial cell migration and tube formation, indicating enhanced angiogenesis. This pro-angiogenic effect was dependent on HIF-1α activation.

**Conclusions:**

We delineate a novel TRIM65-BTG3-HIF-1α signaling axis in OS. TRIM65 degrades BTG3, leading to HIF-1α activation, which drives M2 macrophage polarization and angiogenesis, ultimately fostering tumor progression. Targeting this axis may offer a promising therapeutic strategy for OS.

## Graphical Abstract

**Figure:**
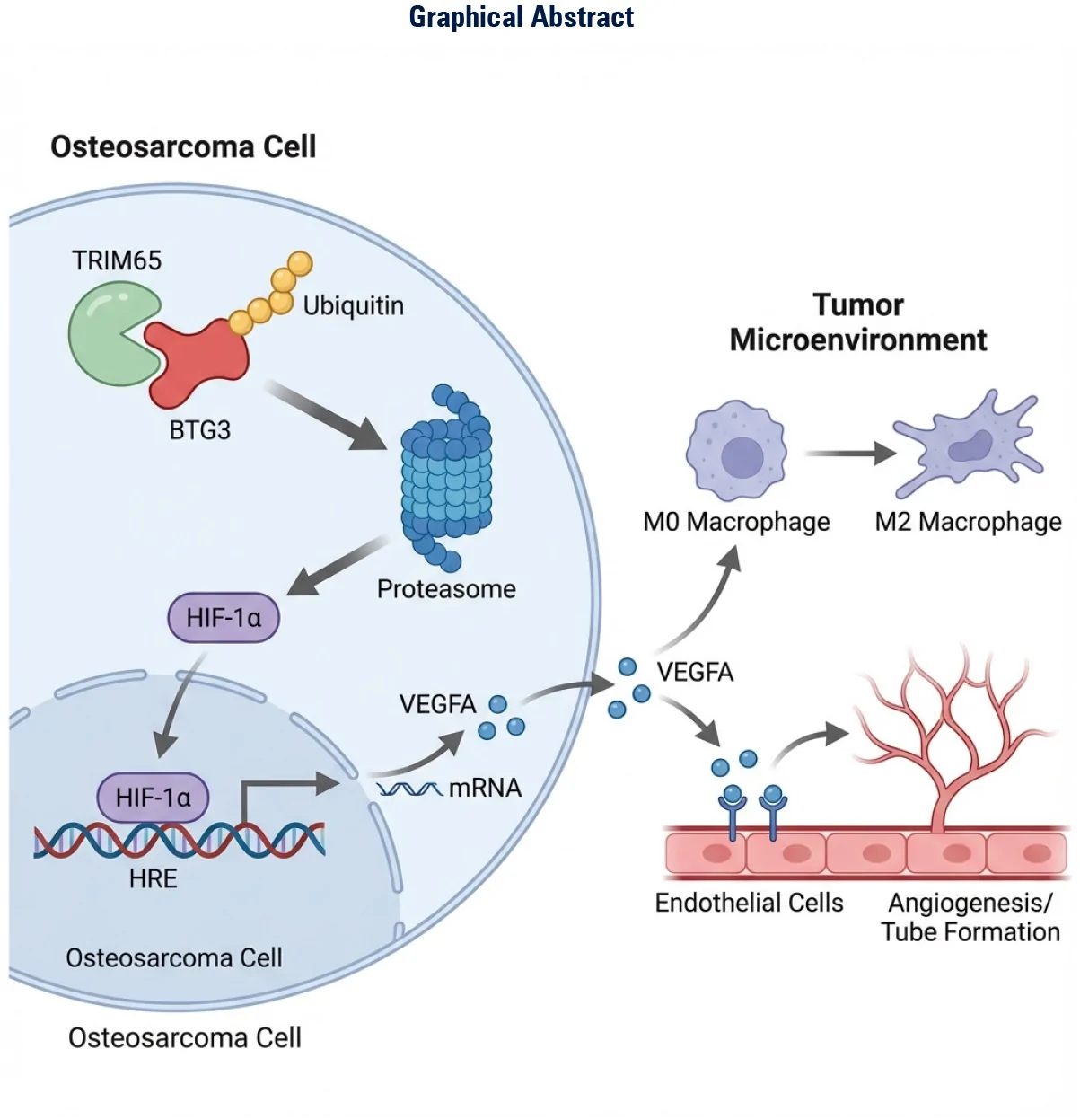

## Introduction

Osteosarcoma (OS) is a highly malignant primary bone tumor, currently commonly seen in children and adolescents in clinical practice. It is characterized by strong invasiveness, early metastasis, and poor prognosis.^[1, 2, 3, 4]^ Research suggests its pathogenesis involves multiple factors including genetic factors, epigenetic regulation, abnormal signaling pathways, and microenvironment interactions.^[5, 6, 7, 8]^ Literature reports indicate that the occurrence of OS is closely related to the inactivation of tumor suppressor genes (such as tumor protein P53 [TP53], retinoblastoma 1 [RB1]), activation of proto-oncogenes (such as myelocytomatosis oncogene [myc], mesenchymal-epithelial transition factor [MET]), and dysregulation of the cell cycle.^[9,10]^ Moreover, the progression of OS is significantly influenced by the tumor microenvironment (TME),^[5,11]^ where interactions among immune cells, stromal cells, and the extracellular matrix play key roles in tumor growth, invasion, and metastasis. ^[11,12]^ The standard clinical treatment model for OS primarily includes surgical resection combined with neoadjuvant and adjuvant chemotherapy.^[13,14]^ Although multidisciplinary comprehensive treatment has significantly improved patient survival rates, the therapeutic efficacy remains unsatisfactory for patients with recurrent or metastatic OS,^[15, 16, 17]^ with literature showing a 5-year survival rate of only 20%-30%.^[9,17]^ Chemotherapy resistance and tumor heterogeneity are the main reasons for treatment failure.^[18,19]^ Therefore, exploring new treatment strategies, particularly targeting the TME and immunotherapy, should be an important direction in OS research.

In recent years, immunotherapy has achieved breakthrough progress in various solid tumors, which also provides new hope for the treatment of OS.^[1,20]^ Despite promising preclinical and early clinical results with immune checkpoint inhibitors, chimeric antigen receptor T (CAR-T) cell therapy, and tumor vaccines in OS,^[21,22]^ significant challenges remain. These include low tumor immunogenicity, an immunosuppressive microenvironment, and treatment-related adverse events.^[12,23]^ Therefore, an in-depth analysis of the immune microenvironment characteristics of OS, particularly the functional status and regulatory mechanisms of immune cells, is of great significance for optimizing immunotherapy strategies.

Tumor-associated macrophages (TAMs) are one of the most important immune cells in the TME,^[24,25]^ possessing high plasticity and functional heterogeneity.^[26]^ Based on their phenotype and function, TAMs can be classified into M1-type (pro-inflammatory) and M2-type (anti-inflammatory).^[27,28]^ M1 macrophages exert anti-tumor effects by secreting pro-inflammatory factors (such as interleukin-12 [IL-12], tumor necrosis factor-α [TNF-α]) and activating anti-tumor immune responses;^[29]^ whereas M2 macrophages support tumor growth and metastasis by secreting immunosuppressive factors (such as IL-10, transforming growth factor-β [TGF-β]) and promoting processes like angiogenesis and tissue remodeling.^[30]^ Studies indicate that the infiltration level of TAMs in OS is closely related to patient prognosis, and therapeutic strategies targeting TAMs (such as colony-stimulating factor 1 receptor (CSF-1R) inhibitors, cluster of differentiation 47 (CD47) blockers) have shown potential efficacy in various tumors.^[30, 31, 32]^

M2 macrophages play a “double-edged sword” role in the TME. On one hand, M2 macrophages directly support tumor growth and metastasis by secreting immunosuppressive factors and promoting angiogenesis;^[33]^ on the other hand, they may indirectly promote tumor progression by regulating tumor cell stemness, metabolic reprogramming, and drug resistance.^[34,35]^ Literature reports that in OS, the infiltration level of M2 macrophages is positively correlated with tumor aggressiveness and metastatic potential, but its specific mechanisms of action are not yet fully elucidated.^[36,37]^ Summarizing current research findings, it can be hypothesized that M2 macrophages may participate in the occurrence and development of OS through the following mechanisms. (1) Immunosuppression: Macrophages may inhibit the activity of T cells and NK cells by secreting factors such as IL-10 and TGF-β, enabling immune evasion. (2) Angiogenesis: Macrophages may promote tumor angiogenesis by secreting factors such as vascular endothelial growth factor (VEGF) and platelet-derived growth factor (PDGF), providing nutritional support for tumor growth.

In summary, immunotherapy for OS still faces significant challenges. We hypothesize that TAMs, particularly M2 macrophages, play an important role in tumor immune evasion and progression. Therefore, this study aimed to investigate the distribution of M2 macrophages in OS and its correlation with patient prognosis. Elucidating the immune microenvironment characteristics of OS may provide a theoretical foundation for developing novel immunotherapeutic strategies and optimizing their combination with existing treatments to improve patient outcomes.

Although the prognostic significance of M2 macrophage infiltration in OS has been recognized, the upstream molecular mechanisms regulating their polarization remain poorly understood. Tripartite motif-containing protein 65 (TRIM65), an E3 ubiquitin ligase, has recently been shown to promote renal cell carcinoma progression through K48-linked ubiquitination and degradation of B-cell translocation gene 3 (BTG3).^[38]^ BTG3, a member of the antiproliferative Tob protein family, functions as a tumor suppressor in various malignancies including renal, prostate, and lung cancers by suppressing RAC-α serine/ threonine-protein kinase [AKT] signaling and maintaining genomic stability.^[39,40]^ Notably, loss of BTG3 has been demonstrated to drive a pro-angiogenic TME through activation of hypoxia-inducible factor-1α (HIF-1α).^[41]^ HIF-1α is a master regulator of hypoxic responses that promotes M2 macrophage polarization and immunosuppressive microenvironment formation.^[42]^ However, the functional involvement of the TRIM65-BTG3-HIF-1α signaling axis in OS and its regulatory role in TME remodeling remain completely unexplored.

## Methods

### Utilization of clinical specimens

This investigation complied with all relevant ethical standards. The animal research protocol received approval from the Animal Ethics Committee of Anhui Medical University, and all procedures adhered to the institution’s guidelines for the humane treatment of laboratory animals. The human subject research component was conducted in accordance with the ethical standards of the institutional research committee (The Clinical Research Ethics Committee of The First Affiliated Hospital of Anhui Medical University) and with the Declaration of Helsinki. Written informed consent was secured from every participant prior to enrollment.

A total of 20 patients diagnosed with OS at the Department of Bone Diseases and Bone Oncology, The First Affiliated Hospital of Anhui Medical University, between January 2021 and December 2024, were included in this study. The inclusion criteria were as follows: (1) patients diagnosed with OS by pathological biopsy; (2) specimen quality meeting experimental requirements (tissue specimen volume ≥ 3 cm^3^); (3) availability of complete clinical data (including age, gender, tumor size, stage, treatment regimen, follow-up data, *etc*.); (4) signed informed consent from the patient or their legal guardian. Exclusion criteria included: (1) unclear pathological diagnosis; (2) specimen quality not meeting experimental requirements (tissue specimen volume < 3 cm^3^); (3) coexistence of severe heart, liver, or kidney dysfunction or other systemic diseases that might affect the study results; (4) Improper specimen preservation or processing not adhering to standard operating procedures (SOPs); (5) incomplete clinical data or inability to obtain follow-up data.

#### Preparation and composition of common solutions

Preparation of tissue fixative: Using a 1 L heat-resistant glass container, 400 mL of PBS buffer was added and heated to 60 °C in a water bath. A total of 20 g paraformaldehyde powder was slowly added to the pre-warmed phosphate buffered saline (PBS) buffer with magnetic stirring for 10 minutes until initial dispersion. Subsequently, 220 μL of 10 mol/L sodium hydroxide *(*NaOH) solution was slowly added to the mixture. Stirring was continued while concentrated hydrochloric acid (HCl) was added dropwise, monitoring the pH change with a pH meter until stabilization at pH 7.2. Finally, PBS buffer was added to bring the total volume to 500 mL. The prepared paraformaldehyde solution was cooled on ice and stored at 4 °C.

### Experimental methods

#### Collection of clinical specimens

Cases meeting the criteria were screened according to the inclusion and exclusion standards. Preoperative physical examination was performed to determine the sampling site. Sterile containers, fixative, liquid nitrogen, and ribonucleic acid (RNA)/deoxyribonucleic acid (DNA) preservation solution were prepared in advance. During surgery, the primary surgeon selected appropriate sampling areas based on tumor location and size, ensuring inclusion of the tumor center, periphery, and adjacent normal tissue. The excised tissue blocks were immediately placed into sterile containers, avoiding compression or damage. As needed, a portion of fresh tissue (approximately 1 cm^3^) was immediately immersed in 4% paraformaldehyde fixative for 24-48 hours for paraffin embedding and pathological examination. Another portion of fresh tissue (approximately 0.5 cm^3^) was rapidly frozen in liquid nitrogen and subsequently transferred to a -80 °C freezer for RNA/ DNA extraction and protein analysis. The remaining tissue was placed in RNA/ DNA preservation solution, stored at 4 °C for 24 hours, and then transferred to a -80 °C freezer. Each container was clearly labeled with patient information (name, medical record number, *etc*.), sampling site, and date. The quantity, size, and macroscopic features (*e.g*., color, texture, necrotic areas) of the sampled tissues were recorded in detail in the laboratory notebook. Tissues in fixative were transported at room temperature, while frozen tissues were transported in liquid nitrogen containers or on dry ice.

#### Preparation of OS tissue paraffin blocks

Tissue specimens fixed in 4% paraformaldehyde were retrieved and rinsed three times with PBS buffer for 10 minutes each. The tissue was trimmed to approximately 1 cm^3^ using a scalpel for subsequent embedding and sectioning. The trimmed tissue was dehydrated through a graded ethanol series: 70% ethanol for 1 hour; 80% ethanol for 1 hour; 95% ethanol for 1 hour; 100% ethanol I for 1 hour; 100% ethanol II for 1 hour. After dehydration, the tissue was sequentially immersed in xylene I and xylene II for 1 hour each. Subsequently, the tissue was immersed in molten paraffin, oriented with the cutting surface facing downward, placed in a pre-warmed embedding mold, and covered with molten paraffin. The mold was then placed on ice to accelerate solidification. Finally, the solidified paraffin block was removed, and its edges were trimmed and labeled with tissue information.

#### Immunofluorescence (IF) staining of tissue sections

Prepared paraffin sections were baked in a 60 °C oven for 30 minutes, followed by sequential immersion in xylene I and xylene II for 10 minutes each. They were then rehydrated through a graded ethanol series (100%, 95%, 80%, 70%) for 5 minutes each. After rinsing in PBS buffer for 5 minutes, sections were immersed in preheated sodium citrate buffer (pH 6.0) and subjected to heat-induced epitope retrieval in a water bath at 95 °C for 15-20 minutes. After cooling to room temperature, sections were washed three times with PBS buffer for 5 minutes each. An appropriate amount of blocking solution (5% bovine serum albumin [BSA] or 10% goat serum) was applied to the sections and incubated at room temperature for 1 hour. The blocking solution was removed, and diluted primary antibodies (anti-inducible nitric oxide synthase [iNOS] and anti-cluster of differentiation 206 [CD206]) were applied separately to completely cover the tissue. Sections were placed in a humidified chamber and incubated overnight at 4 °C (16-18 hours). Unbound primary antibodies were removed by washing three times with PBS buffer for 5 minutes each. Fluorescence-labeled secondary antibodies were applied and incubated at room temperature in the dark for 1 hour. For nuclear staining, sections were washed three times with PBS buffer for 5 minutes each to remove unbound secondary antibodies. 4’, 6-diamidino-2-phenylindole (DAPI) solution (1 μg/mL) was applied and incubated at room temperature in the dark for 10 minutes to label nuclei. Unbound DAPI was removed by washing three times with PBS buffer for 5 minutes each. An anti-fade mounting medium was applied to the sections, covered with a coverslip, and observed under a fluorescence microscope. Corresponding excitation wavelengths were used to detect fluorescence signals from M1-type macrophages, M2-type macrophages, and nuclei.

#### Construction of immune cell infiltration landscape and co-expression network

CIBERSORT is a deconvolution algorithm based on linear support vector regression, used to infer the proportions of immune cell populations in tissue samples by analyzing the expression profiles of 547 signature genes. In this study, we applied the CIBERSORT algorithm to analyze tumor samples from The Cancer Genome Atlas (TCGA) TARGET-OS cohort, quantifying the relative proportions of 22 tumor-infiltrating immune cell types. To ensure result stability, all data were normalized, and the permutation number was set to 1000. Based on the CIBERSORT results, we further screened for 317 genes significantly correlated with M2 macrophages using Pearson correlation analysis (| *r*| > 0.4, *P* < 0.001).

#### Development and validation of a prognostic gene signature

This study constructed a training set using survival and expression data from the TARGET-OS dataset (85 samples, with 59 samples used for training) and employed the GSE21257 dataset as an external validation cohort (53 samples). In the training set, we first screened for M2 macrophage-associated genes related to prognosis using univariate Cox regression analysis. Subsequently, the least absolute shrinkage and selection operator (LASSO) regression algorithm (with iterations set to 1E6) was employed for feature selection. An optimal subset of prognostic genes was identified through iterative methods to construct an M2 macrophage-related gene (MRG) signature. Based on the LASSO results, a multivariate Cox regression model was further used to determine the final set of genes included in the signature.

According to the multivariate Cox regression analysis results, the risk score for each patient was calculated using the following formula: Risk score = Σ(coef[b] × EXP[b]), where b represents the expression value of a gene and coef(b) is its corresponding regression coefficient. Using the median risk score as the cutoff, patients were stratified into high-risk and low-risk groups. Kaplan-Meier (K-M) survival analysis was performed to compare clinical prognosis differences between the two groups. The predictive accuracy of the model was assessed using receiver operating characteristic (ROC) curves and time-dependent C-index. Furthermore, stratified analysis was conducted to evaluate the independent prognostic value of the M2 macrophage-related risk score (MRS).

#### Validation and clinical application of the MRS

In the external validation set, patients were stratified into high-risk and low-risk groups using the same risk score grouping method as in the training set (median risk score as cutoff). Differences in survival rates between the two groups were assessed using K-M survival analysis and risk curves. Additionally, to further validate the clinical significance of the risk score, stratified analysis was performed based on age, gender, and metastasis status. K-M survival analysis was conducted separately for high-risk and low-risk groups within each stratum to investigate differences in clinical prognosis.

#### Construction and evaluation of a nomogram

A nomogram was constructed by integrating the risk score with clinicopathological factors (such as age, gender, race, and metastasis status) to predict the 1-year, 3-year, and 5-year overall survival (OS) rates for OS patients. To validate the predictive accuracy of the nomogram, calibration curves were used to compare the observed OS rates with those predicted by the nomogram. The calibration curves graphically illustrate the consistency between predicted and observed values, further assessing the model’s reliability.

#### mRNA sequencing and differential analysis of OS and adjacent tissues

In this study, freshly collected OS and adjacent normal tissues were used for mRNA sequencing to investigate mRNA expression profiles in OS samples. Both OS and adjacent control groups were stored in liquid nitrogen after collection, each containing three independent biological replicates. The sequencing work was performed by Annoroad Gene Technology Co., Ltd. (Beijing). After sequencing completion, significantly differentially expressed genes between the two groups were identified with specific parameters set as fold change > 1.5 and *P* < 0.05. Additionally, functional enrichment analysis was performed based on the mRNA sequencing results.

#### Data-independent acquisition (DIA) quantitative proteomics and differential analysis of OS and adjacent tissues

In this study, freshly collected OS and adjacent normal tissues were used for DIA quantitative proteomics to investigate protein expression profiles in OS samples. Three pairs of OS and adjacent control tissues were stored in liquid nitrogen after collection, each containing three independent biological replicates. The proteomics analysis was performed by Ning’an Life Sciences Co., Ltd. (Shanghai). After analysis, the threshold for differential expression was set at a fold change of 2 with a significance *P*-value of 0.05. Protein domain enrichment analysis was conducted on the significantly differentially expressed proteins identified.

#### Co-immunoprecipitation (Co-IP) assays

Cells were lysed in ice-cold IP lysis buffer (50 mmol/ L Tris-HCl pH 7.4, 150 mmol/ L sodium chlorid [NaCl], 1% nonidet P-40 [NP-40], 0.5% sodium deoxycholate, 0.1% sodium dodecyl sulfate [SDS]) supplemented with protease inhibitor cocktail and 10 mmol/L N-ethylmaleimide (NEM) to inhibit deubiquitination. Lysates were centrifuged at 12,000×*g* for 15 min at 4 °C, and supernatants were incubated with primary antibodies (anti-myc, antihemagglutinin [HA], or anti-BTG3) or control IgG overnight at 4 °C, followed by addition of Protein A/ G magnetic beads for 2 hours. Immunoprecipitates were washed four times with lysis buffer, eluted in 2× SDS loading buffer, and analyzed by Western blotting (WB).

#### Quantitative real-time polymerase chain reaction (qRT-PCR)

Total RNA was extracted from cells or tissues using TRIzol reagent according to the manufacturer’s instructions. RNA purity and concentration were assessed by spectrophotometry. First-strand cDNA was synthesized from 1 μg of total RNA using the PrimeScript RT Reagent Kit (Vazyme, China) following the manufacturer’s protocol. Quantitative real-time PCR was performed using SYBR Green Master Mix (Vazyme, China) on a LightCycler 480 System. The PCR reaction mixture (20 μL) contained 10 μL of SYBR Green Master Mix, 0.5 μL of each primer (10 μmol/L), 2 μL of cDNA template, and 7 μL of nuclease-free water. The thermal cycling conditions were as follows: initial denaturation at 95°C for 5 minutes, followed by 40 cycles of 95 °C for 15 seconds, 60 °C for 30 seconds, and 72 °C for 30 seconds. Melting curve analysis was performed to confirm specific amplification.

Gene expression levels were normalized to the housekeeping gene GAPDH and calculated using the 2^(-ΔΔCt) method. All reactions were performed in triplicate, and the results are expressed as mean ± standard deviation (SD). The primer sequences used in this study are listed. BTG3-F: 5’-ATGAAATTGCTGCCGTTGTCT-3’; BTG3-R: 5’-GCCTGTCCTTTCGATGGTTTT-3’; TRIM65-F: 5’-CGCCAACCGTCACTTCTATCT-3’; TRIM65-R: 5’-ACAGGGTCAGGGTCCTACC-3’.

### Statistical analysis

#### Bioinformatics and survival analysis

##### Immune cell infiltration analysis

The CIBERSORT algorithm was applied to RNA-seq data from the TCGA TARGET-OS cohort to quantify the relative proportions of 22 tumor-infiltrating immune cell types. This deconvolution algorithm uses linear support vector regression based on the expression profiles of 547 signature genes. To ensure result stability, the permutation number was set to 1000.

##### Correlation analysis

Pearson correlation analysis was performed to identify genes significantly associated with M2 macrophage infiltration levels. Genes with |*r*| > 0.4 and *P* < 0.001 were considered significantly correlated. A total of 317 genes were identified as significantly related to M2 macrophages.

##### Prognostic gene screening

In the training set (59 samples from TARGET-OS cohort), univariate Cox regression analysis was performed to screen for M2 MRGs with potential prognostic value, identifying 27 candidate genes. To refine the signature and avoid overfitting, the LASSO regression algorithm was employed for feature selection with iterations set to 1×106. Subsequent multivariate Cox regression analysis yielded a final prognostic model comprising 7 genes (*FPR1*, *BTG3*, *DAB2*, *ARHGAP25*, *CCL8*, *CTTNBP2NL*, *CREBL2*).

##### Risk score calculation and stratification

According to the multivariate Cox regression analysis results, the risk score for each patient was calculated using the following formula: Risk score = Σ(coef[b] × EXP[b]), where b represents the expression value of a gene and coef(b) is its corresponding regression coefficient. Using the median risk score as the cutoff, patients were stratified into high-risk and low-risk groups.

##### Survival analysis

K-M survival analysis was performed to compare OS between the high-risk and low-risk groups. The predictive accuracy of the M2 MRS was assessed using ROC curves for 1-year, 3-year, and 5-year OS predictions. Concordance index (C-index) was calculated to evaluate the discriminative ability of the model. Calibration curves were generated to assess the agreement between predicted and observed survival probabilities.

##### Subgroup and validation analysis

Stratified analysis was conducted to evaluate the independent prognostic value of the MRS across different patient subgroups based on age (≤ 65 years), gender, and metastasis status. The prognostic model was validated in an internal validation cohort from the TARGET-OS dataset.

#### Experimental data analysis

##### Quantitative data analysis

For comparisons between two groups (*e.g*., tumor *vs*. adjacent normal tissue, control vs. knockdown), Student’s *t*-test was used after confirming normal distribution of data using the Shapiro-Wilk test. For multiple group comparisons (*e.g*., different cell lines or treatment groups), one-way analysis of variance (ANOVA) followed by Tukey’s post-hoc test was performed. Homogeneity of variance was assessed using Levene’s test.

##### qRT-PCR data analysis

Relative gene expression levels were calculated using the 2^(-ΔΔCT)^ method, with *GAPDH* serving as the internal reference gene. The ΔCT value was calculated as (CT_target gene - CT_*GAPDH*), and ΔΔCT was determined by comparing ΔCT values between experimental and control groups. The control group was normalized to a value of 1.

##### IF quantification

For macrophage infiltration analysis, five random fields per sample were captured at high magnification. Positive cells were counted manually by two independent investigators in a blinded manner. Data represent mean ± SD from five random fields.

##### Functional assay quantification

For colony formation, transwell migration/invasion, tube formation, and wound healing assays, quantitative data were obtained from at least three independent experiments performed in triplicate. Cell counts or relative areas were measured using ImageJ software (version 1.53). Statistical significance was determined using Student’s *t*-test or ANOVA as appropriate.

##### *In vivo* tumor growth analysis

Tumor volumes were calculated using the formula V = (length × width^2^)/2. Tumor growth curves were analyzed using two-way ANOVA with repeated measures, followed by Sidak’s multiple comparisons test. Final tumor weights were compared using Student’s *t*-test.

## Results

### M2 macrophages are predominant in the OS microenvironment

To characterize the immune landscape of OS, we began by examining clinical OS specimens. Histological analysis using hematoxylin and eosin (H&E) staining revealed that OS tissues were characterized by significant infiltration of irregular, mononuclear cells with reniform or oval nuclei, clustered or diffusely distributed within the tumor stroma and between tumor cell nests, a morphology consistent with macrophages (Figure 1A).

**Figure 1:**
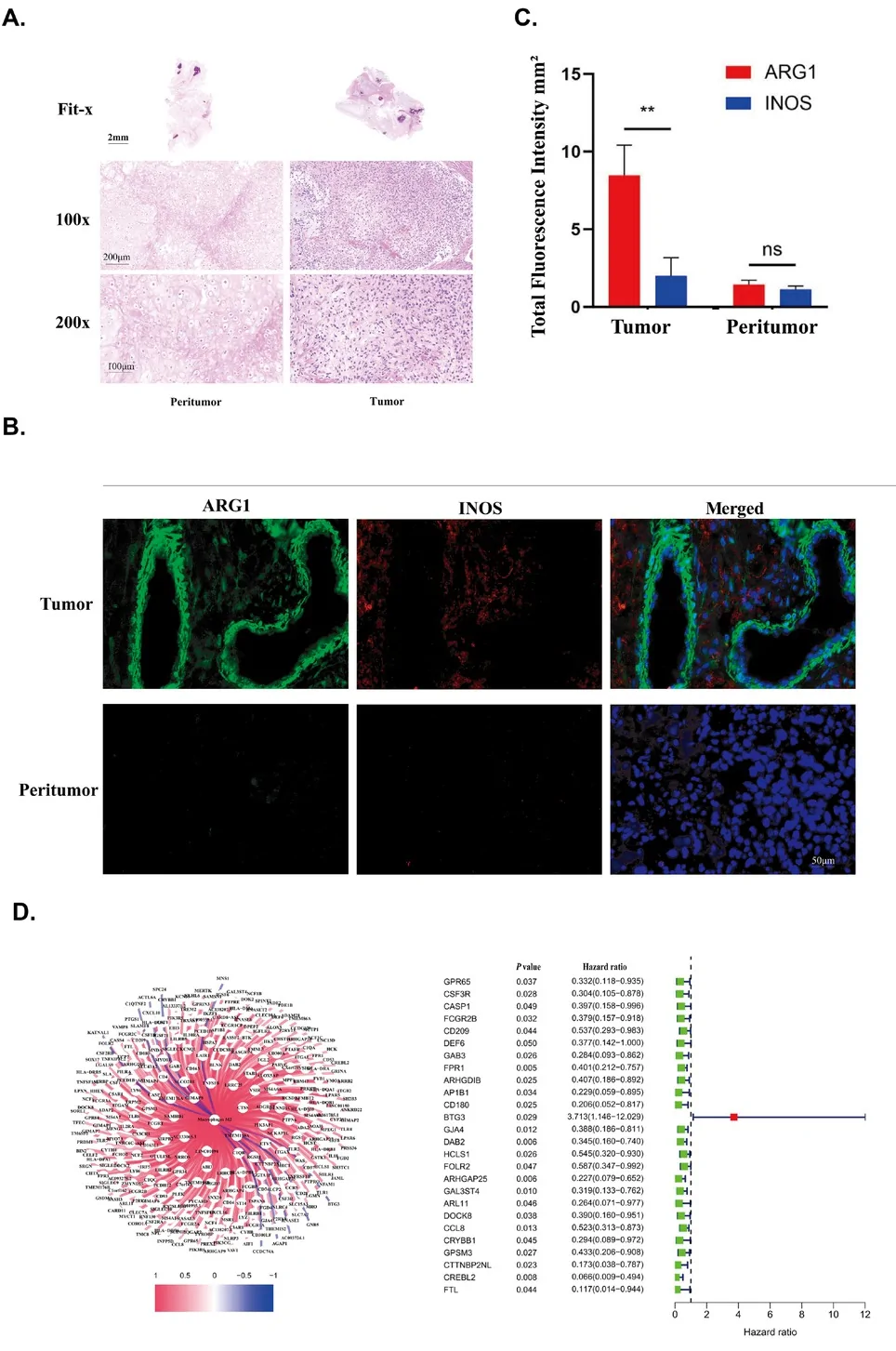
Identification of significant macrophage infiltration with M2 phenotype dominance in the OS TME. (A) Representative H&E staining of OS tissue, showing morphological features indicative of significant macrophage infiltration within the tumor stroma. (B) IF staining for M1 (iNOS+, red) and M2 (ARG1+, green) macrophage markers in OS and paired adjacent normal tissues. Nuclei were counterstained with DAPI (blue). Scale bar: 50 μm. (C) Quantitative analysis of M1 and M2 macrophage infiltration based on IF signals. Data are presented as mean ± SD (*n* = 5 random fields per sample). ^**^P < 0.01 *vs*. adjacent normal tissue, analyzed by Student’s *t*-test. M2 macrophages were significantly predominant in the OS microenvironment. (D) CIBERSORT algorithm analysis and Pearson correlation analysis: Pearson correlation analysis identified a total of 317 genes related to M2 macrophages and 27 genes related to M2 macrophages were identified as potential prognostic indicators. H&E: hematoxylin and eosin; SD: standard deviation; TME: tumor microenvironment; IF: immunofluorescence; OS, osteosarcoma; iNOS: inducible nitric oxide synthase; ARG1: arginase 1; TCGA-OS: The Cancer Genome Atlas Osteosarcoma.

We then performed IF staining to precisely identify and quantify macrophage subtypes. Staining for the M1 marker iNOS (red) and the M2 marker arginase 1 (ARG1; green) on paired OS and adjacent normal tissues confirmed a substantial increase in total macrophage infiltration in OS. High-magnification showed that ARG1-positive cells were widely distributed in the tumor stroma, with an obvious aggregation trend at the tumor-stroma junction. Critically, quantitative analysis of five random fields demonstrated that M2 macrophages were significantly more abundant than M1 macrophages within OS tissues (Figure 1B, C).

Notably, ARG1 was selected as the M2 polarization marker for tissue IF staining due to its established reliability in formalin-fixed paraffin-embedded (FFPE) tissue sections and its strong correlation with M2 functional phenotype in OS microenvironment characterization. For *in vitro* THP-1-derived macrophage polarization experiments, CD206 (mannose receptor) was employed as the primary M2 marker, consistent with established protocols for flow cytometry-based quantification of alternatively activated macrophages. Both markers are well-validated indicators of M2 polarization, and their differential use reflects methodological optimization for distinct experimental contexts rather than biological inconsistency.

### Construction and validation of an M2 macrophage-related prognostic signature

Given the dominance of M2 macrophages, we sought to define their molecular influence on OS progression. We applied the CIBERSORT algorithm to RNA-seq data from the TARGET-OS cohort to quantify immune cell infiltration. Pearson correlation analysis identified 317 genes whose expression levels were significantly correlated with M2 macrophage abundance (|*r*| > 0.4, *P* < 0.001; Figure 1D).

Univariate Cox regression analysis of these genes within the TARGET-OS training set identified 27 M2 MRGs with potential prognostic value (Figure 2A). To refine this signature and avoid overfitting, we employed the LASSO regression algorithm, which helped select an optimal prognostic gene subset (Figure 2B). Subsequent multivariate Cox regression analysis yielded a final prognostic model comprising 7 genes (*FPR1*, *BTG3*, *DAB2*, *ARHGAP25*, *CCL8*, *CTTNBP2NL*, *CREBL2*). Patients were assigned a risk score based on this 7-gene signature. Kaplan-Meier survival analysis revealed that patients in the high-risk group had a significantly shorter OS compared to the low-risk group in the training set (*P* < 0.05; Figure 2C). This prognostic value was confirmed in an internal validation cohort (*P* < 0.05; Figure 2D). The predictive accuracy of the M2 MRS was evaluated by ROC curves, which showed area under the curve (AUC) values of 0.784, 0.766, and 0.742 for predicting 1, 3, and 5-year survival, respectively (Figure 2E). Furthermore, the MRS demonstrated superior predictive power compared to other clinical pathological variables (Figure 2F).

**Figure 2:**
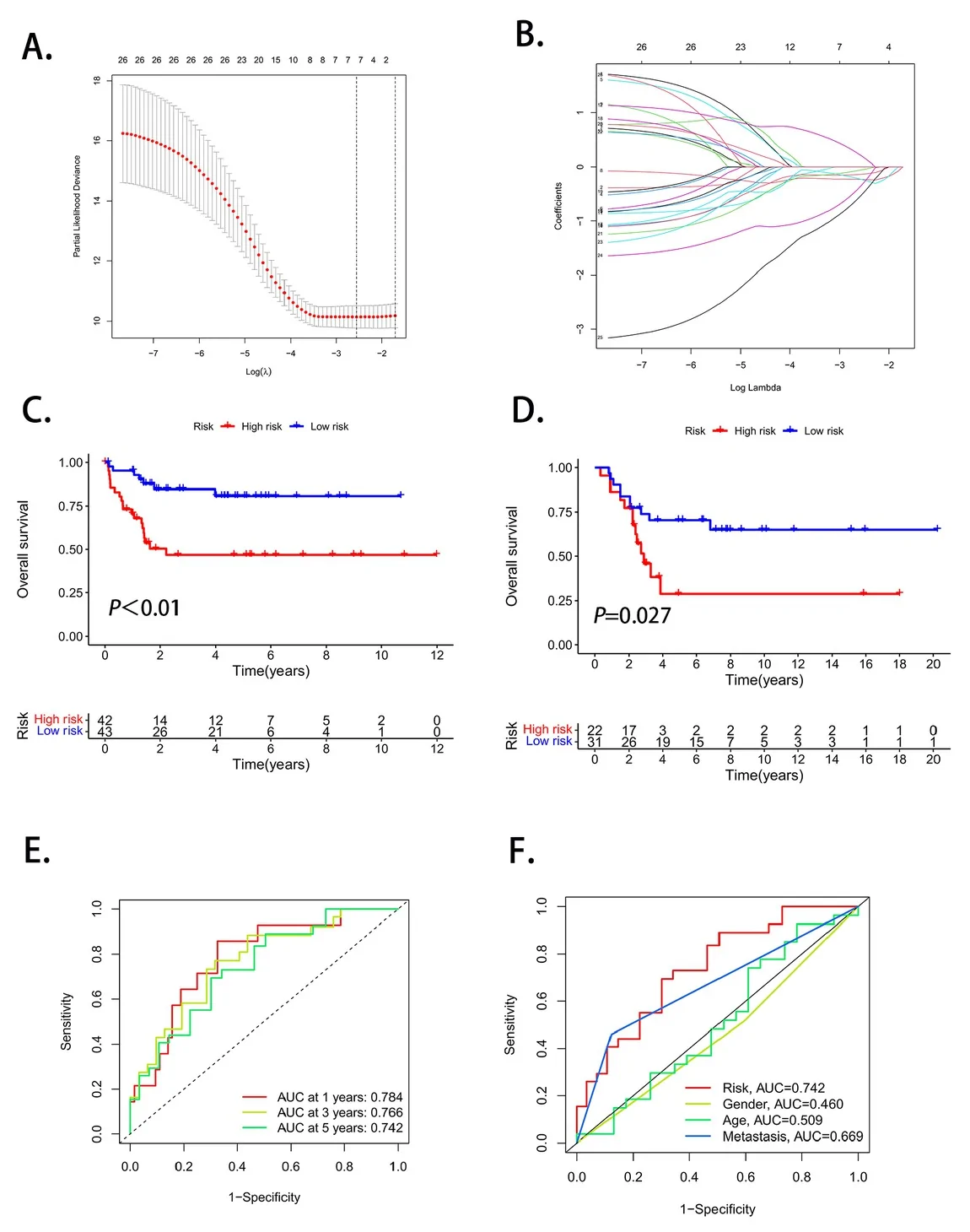
**Construction and validation of an M2 macrophage-related prognostic signature for OS. (A) LASSO cross-validation curve for selecting the optimal tuning parameter (λ). (B) LASSO coefficient profiles of the 27 candidate prognostic genes identified by univariate Cox regression. (C) Kaplan-Meier survival analysis of the high-risk and low-risk groups stratified by the MRS in the training set (TARGET-OS cohort). Patients in the high-risk group had significantly poorer OS** (***P* < 0.05). (D) Kaplan-Meier survival analysis in the internal validation set, confirming the prognostic value of the MRS. (E) ROC curves demonstrating the predictive accuracy of the MRS for 1-year, 3-year, and 5-year OS (AUC values: 0.784, 0.766, 0.742, respectively). (F) ROC curve comparing the predictive power of the MRS with other clinical pathological variables. ROC: receiver operating characteristi; MRGs: macrophage-related genes; MRS: macrophage-related risk score; AUC: area under the curve; OS: overall survival; OS: osteosarcoma; ROC, receiver operating characteristic; MRS: macrophage-related risk score.**

### Clinical translation of the prognostic model and identification of BTG3 as a key player

To facilitate clinical application, we constructed a nomogram that integrated the MRS with key clinical factors (age, gender, race, metastasis status) to predict the probability of 1-, 3-, and 5-year OS for individual patients (Figure 3A).

**Figure 3:**
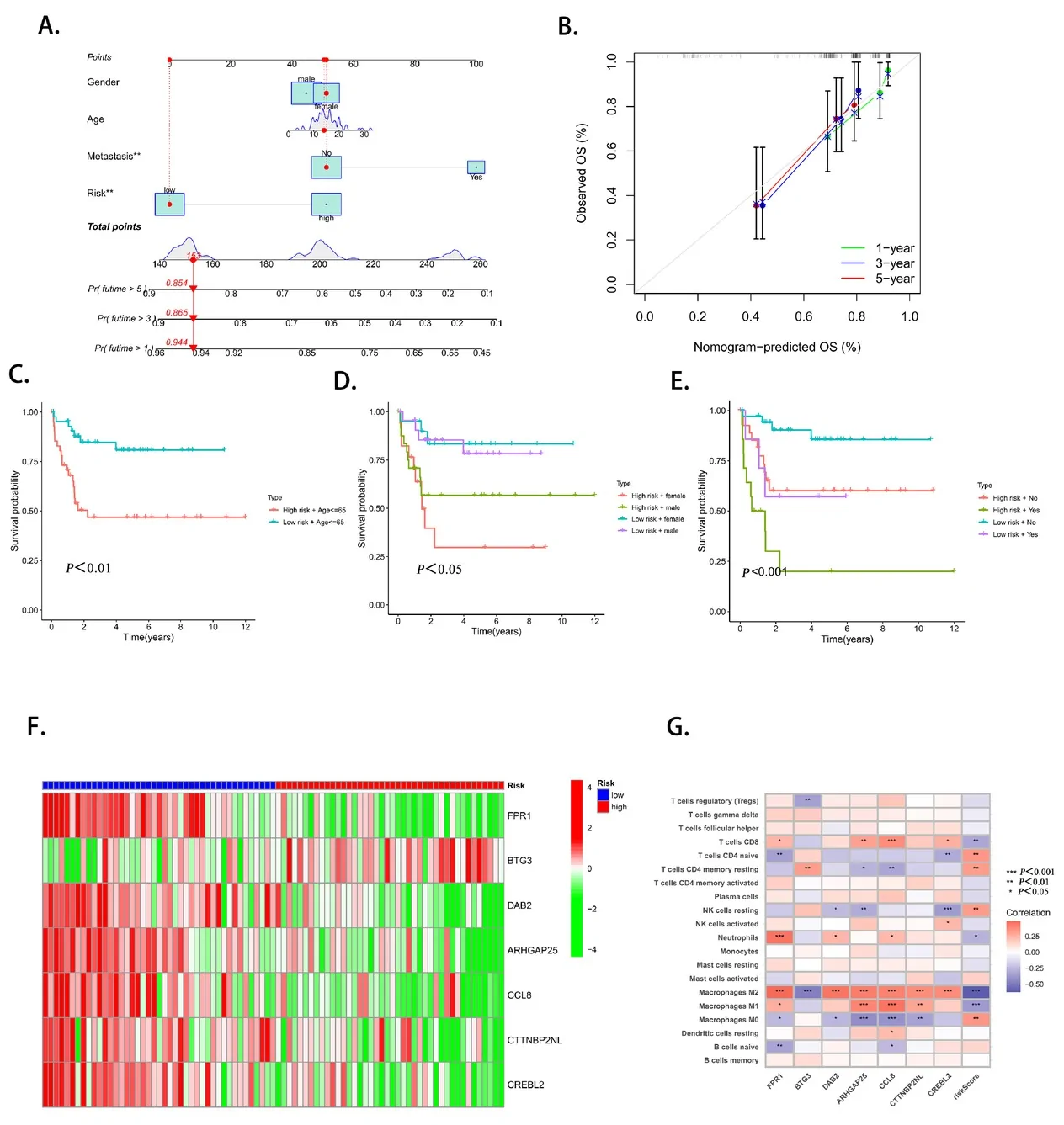
Clinical translation of the prognostic model and identification of BTG3 as a key gene with clinical significance. (A) A nomogram integrating the MRS with clinical factors (age, gender, race, metastasis) to predict 1-, 3-, and 5-year OS probability for OS patients. (B) Calibration curves of the nomogram, showing good agreement between predicted and observed survival probabilities. (C-E) Subgroup survival analysis based on (C) age (≤65 years), (D) gender, and (E) metastasis status, demonstrating the consistent prognostic value of the MRS across different patient subgroups. (F) Heatmap of the expression patterns of the 7-model genes in the TCGA-OS cohort. BTG3 showed the most significant differential expression and was upregulated in almost all high-risk patient samples. (G) Immune cell correlation analysis for the seven model genes. BTG3 expression showed a significant negative correlation with M2 macrophage infiltration (*P* < 0.05), while the other six genes were positively correlated. OS: overall survival; MRS: macrophage-related risk score; OS: osteosarcoma; TCGA: The Cancer Genome Atlas. ^*^P < 0.05, ^**^P < 0.01, ^***^P < 0.001.

The calibration curves for the nomogram showed excellent agreement between the predicted and observed survival probabilities (Figure 3B). Subgroup analyses confirmed the consistent prognostic performance of the MRS across patients stratified by age (≤65 years), gender, and metastasis status (Figure 3C-E). Analysis of the expression patterns of the 7 model genes in the TCGA-OS cohort *via* heatmap highlighted that BTG3 exhibited the most significant differential expression and was upregulated in almost all high-risk patient samples (Figure 3F). Interestingly, immune cell correlation analysis indicated that while the other six genes showed a positive correlation with M2 macrophage infiltration, BTG3 expression displayed a significant negative correlation (*P* < 0.05; Figure 3G).

### BTG3 exhibits an up-transcription-down-translation pattern and is an independent prognostic factor

The consistent upregulation of BTG3 mRNA in high-risk patients prompted us to investigate its protein expression. WB analysis of 20 paired fresh clinical OS specimens revealed that BTG3 protein levels were significantly lower in tumor tissues compared to their adjacent normal counterparts (Figure 4A).

**Figure 4:**
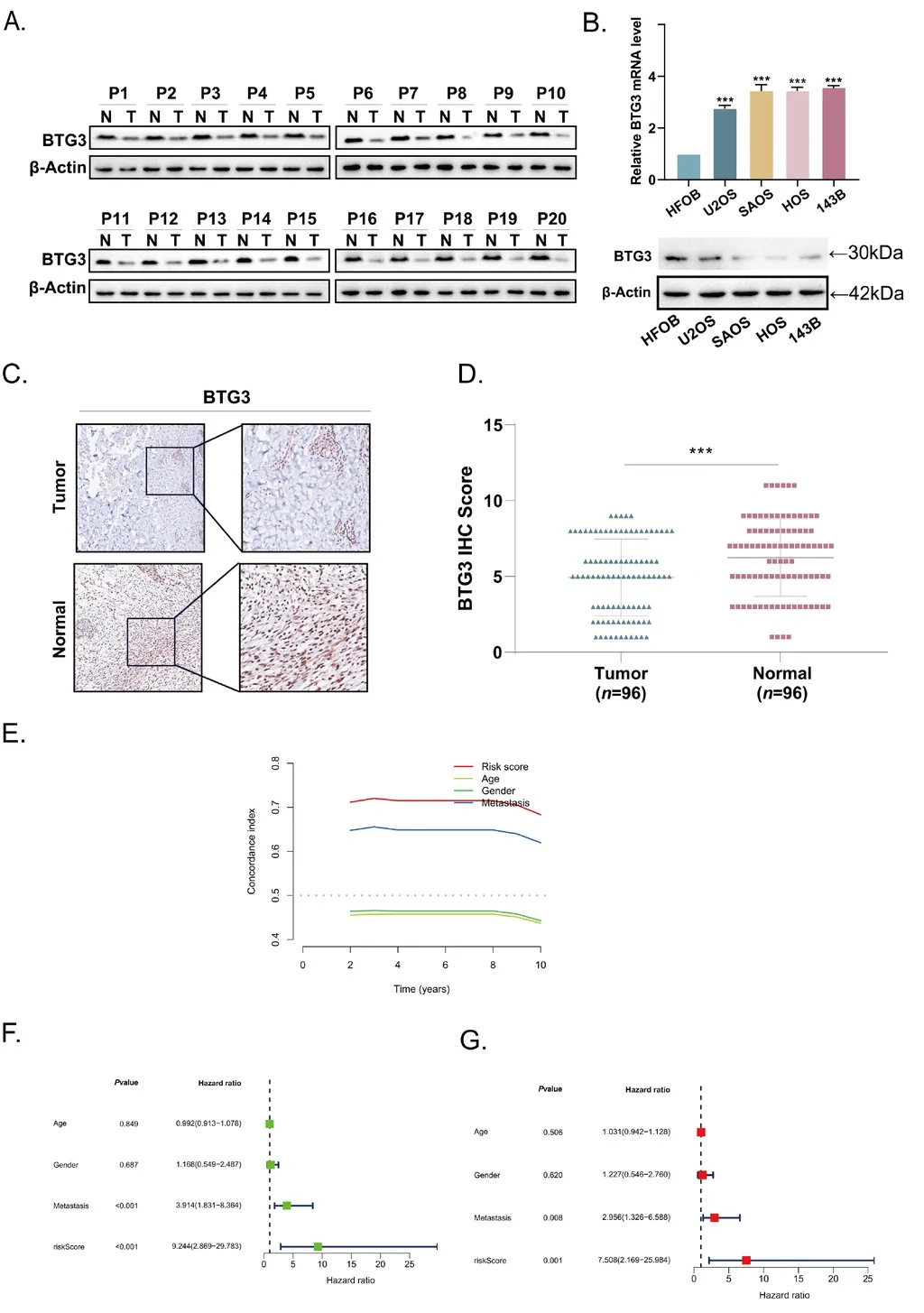
BTG3 exhibits a high-transcription-low-translation pattern and serves as an independent prognostic factor in OS. (A)The results of the WB experiment on clinical specimens showed that BTG3 protein was underexpressed in OS. (B)The qPCR and WB experiments on OS cell lines showed that BTG3 exhibits high transcription and low expression in OS cell lines. (C, D) Adjacent normal bone tissue shows strong positive expression of BTG3 protein. The paired OS tissue shows markedly absent or reduced BTG3 protein expression. (E) C-index analysis comparing the prognostic power of the MRS with other clinical variables. (F) Forest plot from univariate Cox regression analysis, identifying BTG3 high expression as a significant risk factor. (G) Forest plot from multivariate Cox regression analysis, confirming BTG3 as an independent prognostic factor for OS patient survival. OS: osteosarcoma; hFOB: human fetal osteoblast; U2OS: U-2 osteosarcoma; SAOS: sarcoma osteogenics; HOS: human osteosarcoma; IHC: immunohistochemistry; MRS: macrophage-related risk score; WB, western blot; BTG3: B cell translocation gene 3; qPCR: quantitative real-time polymerase chain reaction. ^***^P < 0.001.

This paradoxical “up-transcription-down-translation” phenomenon was further supported by our transcriptomic and proteomic profiling of clinical samples. mRNA sequencing of 5 patient pairs identified BTG3 among 2857 significantly upregulated transcripts (Supplementary Figure S1A), while DIA quantitative proteomics of 3 patient pairs confirmed BTG3 as one of the most significantly downregulated proteins in OS tissues (Supplementary Figure S1C). This pattern was recapitulated *in vitro*. qRT-PCR and WB analysis of multiple OS cell lines (U2OS, HOS, SAOS, 143B) and the normal osteoblastic cell line hFOB confirmed that BTG3 mRNA was highly transcribed, while its protein level was significantly downregulated in OS cells (Figure 4B). Immunohistochemistry (IHC) on a tissue microarray containing 96 cases further validated the marked reduction or absence of BTG3 protein staining in OS tissues compared to the strong positive signal observed in adjacent normal bone (Figure 4C, D). The C-index analysis indicated that the MRS had strong prognostic power compared to other clinical variables (Figure 4E). Univariate Cox regression analysis identified high BTG3 mRNA expression as a significant risk factor for poor survival (Figure 4F). Importantly, multivariate Cox regression analysis confirmed that BTG3 is an independent prognostic factor for OS patient survival (Figure 4G).

### The E3 ubiquitin ligase TRIM65 promotes OS malignancy by mediating ubiquitin-dependent degradation of BTG3

To elucidate the mechanism behind the low BTG3 protein levels, we analyzed DIA quantitative proteomics data from OS tissues. This screen identified TRIM65, an E3 ubiquitin ligase, as one of the most significantly upregulated proteins (Supplementary Figure S2A). Functional classification suggested enrichment in processes like post-translational modification (Supplementary Figure S2B). We hypothesized that TRIM65 targets BTG3 for degradation. Indeed, knocking down TRIM65 in U2OS cells led to a marked increase in BTG3 protein levels (Figure 5A). Co-IP assays demonstrated that TRIM65 directly interacts with BTG3 and promotes its polyubiquitination (Figure 5B). TRIM65 was confirmed to be highly expressed at both mRNA and protein levels in multiple OS cell lines compared to hFOB cells (Figure 5C). Efficient knockdown of TRIM65 was achieved using two specific short hairpin RNAs (shRNAs; shTRIM651, shTRIM652), as verified by qRT-PCR and WB (Figure 5D, E). Functional assays showed that TRIM65 knockdown significantly impaired the clonogenic ability (Figure 5F), migration (Figure 5G), and invasion (Figure 5H) of OS cells *in vitro*. To further confirm the oncogenic role of TRIM65, we performed gain-of-function experiments by overexpressing TRIM65 in U2OS cells (which have relatively low endogenous TRIM65 levels). Ectopic expression of TRIM65 significantly enhanced cell migration and invasion capabilities compared to vector control (Supplementary Figure S3A, B), corroborating the knockdown results and establishing TRIM65 as a bona fide promoter of OS malignancy. Furthermore, in a mouse xenograft model, TRIM65 knockdown significantly suppressed tumor growth, as evidenced by smaller tumor volumes and lower final tumor weights (Supplementary Figure S4A-D).

**Figure 5:**
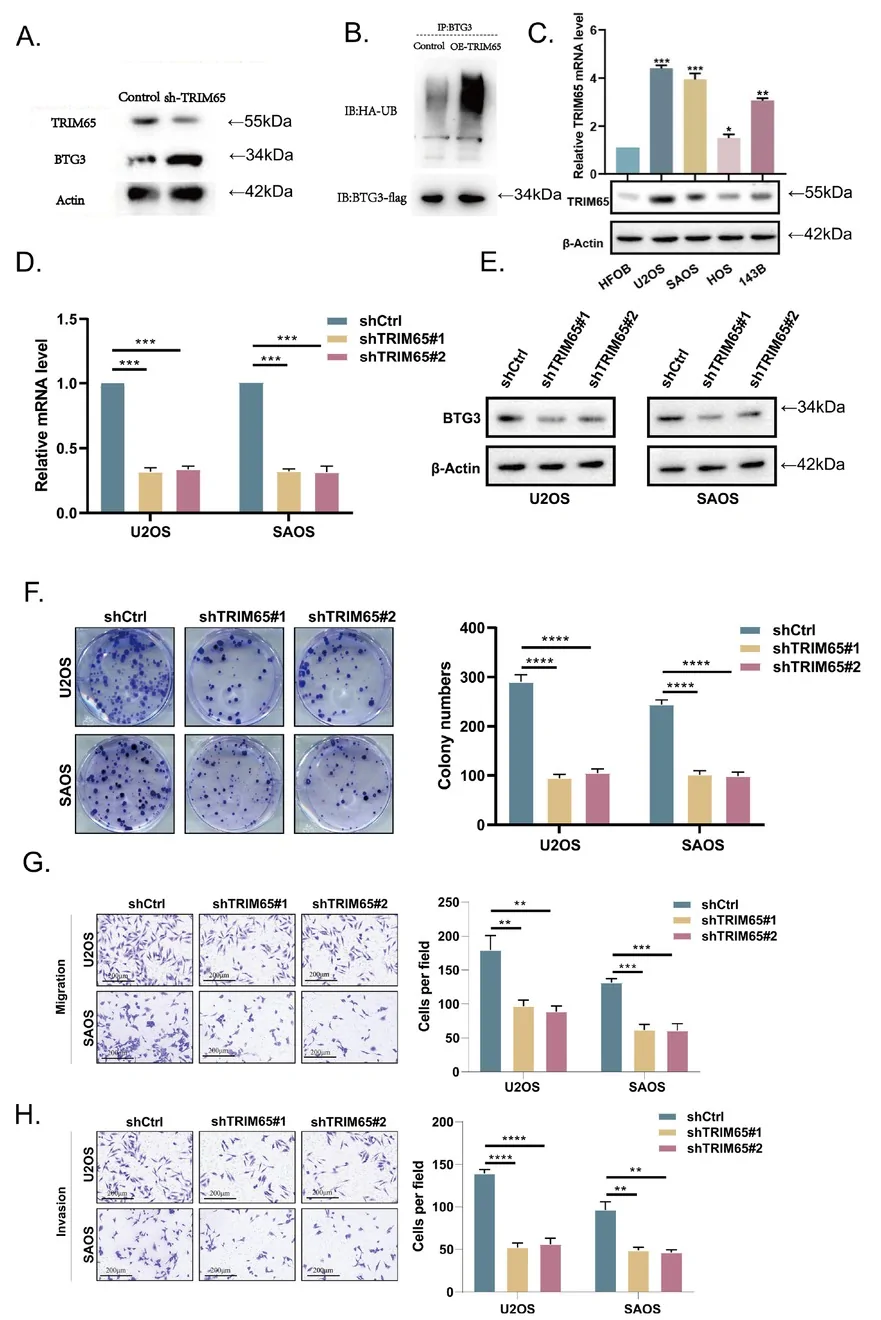
The E3 ubiquitin ligase TRIM65 promotes OS malignancy by mediating ubiquitin-dependent degradation of BTG3. (A) WB analysis showing that knockdown of TRIM65 (shTRIM65) in U2OS cells led to a marked increase in BTG3 protein levels compared to the control (shCtrl) group. (B) Immunoprecipitation and ubiquitination assays. Myc-tagged BTG3 was immunoprecipitated, and ubiquitination was detected with an HA-ubiquitin antibody. Co-expression of TRIM65 significantly enhanced the polyubiquitination of BTG3, indicating TRIM65 acts as an E3 ubiquitin ligase for BTG3. (C) (Top) WB analysis confirming high protein expression of TRIM65 in multiple OS cell lines (U2OS, 143B, SAOS, HOS) compared to the normal osteoblastic cell line hFOB. (Bottom) qRT-PCR analysis showing corresponding high mRNA expression levels of TRIM65 in OS cell lines. Data are mean ± SD. (D) qRT-PCR validation of TRIM65 knockdown efficiency at the mRNA level in OS cells transfected with shCtrl, shTRIM65#1, and shTRIM65#2. (E) WB analysis confirming efficient knockdown of TRIM65 at the protein level in the corresponding cell groups. (F) Colony formation assays. Representative images (left) and quantitative analysis (right) showing that TRIM65 knockdown significantly impaired the clonogenic ability of OS cells. (G)Transwell migration assays without matrigel. Representative images (left) and quantitative analysis (right) demonstrating that TRIM65 knockdown significantly reduced the number of migrating cells. (H) Transwell invasion assays with Matrigel. Representative images (left) and quantitative analysis (right) showing that TRIM65 knockdown markedly inhibited the invasive capacity of OS cells. *n* = 3. ^*^P < 0.05, ^**^P < 0.01, ^***^P < 0.001, ^****^P < 0.0001. OS: osteosarcoma; SD: standard deviation; qRT-PCR: quantitative real-time reverse transcription polymerase chain reaction; BTG3: B cell translocation gene 3; TRIM65: tripartite motif-containing 65; hFOB: human fetal osteoblast; U2OS: U-2 osteosarcoma; SAOS: sarcoma osteogenics; HOS: human osteosarcoma; myc: myelocytomatosis oncogen; mRNA: messenger ribonucleic acid; WB: western blot.

### BTG3 deficiency activates the HIF-1α signaling pathway

Having established that TRIM65-mediated degradation leads to BTG3 protein loss in OS, we next sought to investigate the functional consequences of BTG3 deficiency. Previous studies have reported that BTG3 can regulate HIF-1α in other cellular contexts. Given the critical role of HIF-1α in TME remodeling, particularly in promoting angiogenesis and immune modulation, we hypothesized that BTG3 loss in OS might lead to HIF-1α activation.

To investigate the molecular mechanism by which BTG3 regulates HIF-1α signaling in OS, we performed luciferase reporter assays using both the COX-2 promoter and a synthetic hypoxia response element (HRE) reporter.

We first examined the effect of HIF-1α overexpression and BTG3 co-expression on COX-2 transcriptional activity. As shown in Figure 6A, under normoxic conditions, transfection of HA-HIF-1α alone increased Cox-2 promoter-driven luciferase activity, and this effect was further enhanced under hypoxia. However, co-transfection of myc-BTG3 with HA-HIF-1α significantly suppressed HIF-1α-induced Cox-2 promoter activation under both normoxic and hypoxic conditions, suggesting that BTG3 negatively regulates HIF-1α-mediated transcription. WB analysis confirmed the expression of HA-HIF-1α and myc-BTG3 proteins under the corresponding experimental conditions, with Actin serving as a loading control (Figure 6B).

**Figure 6:**
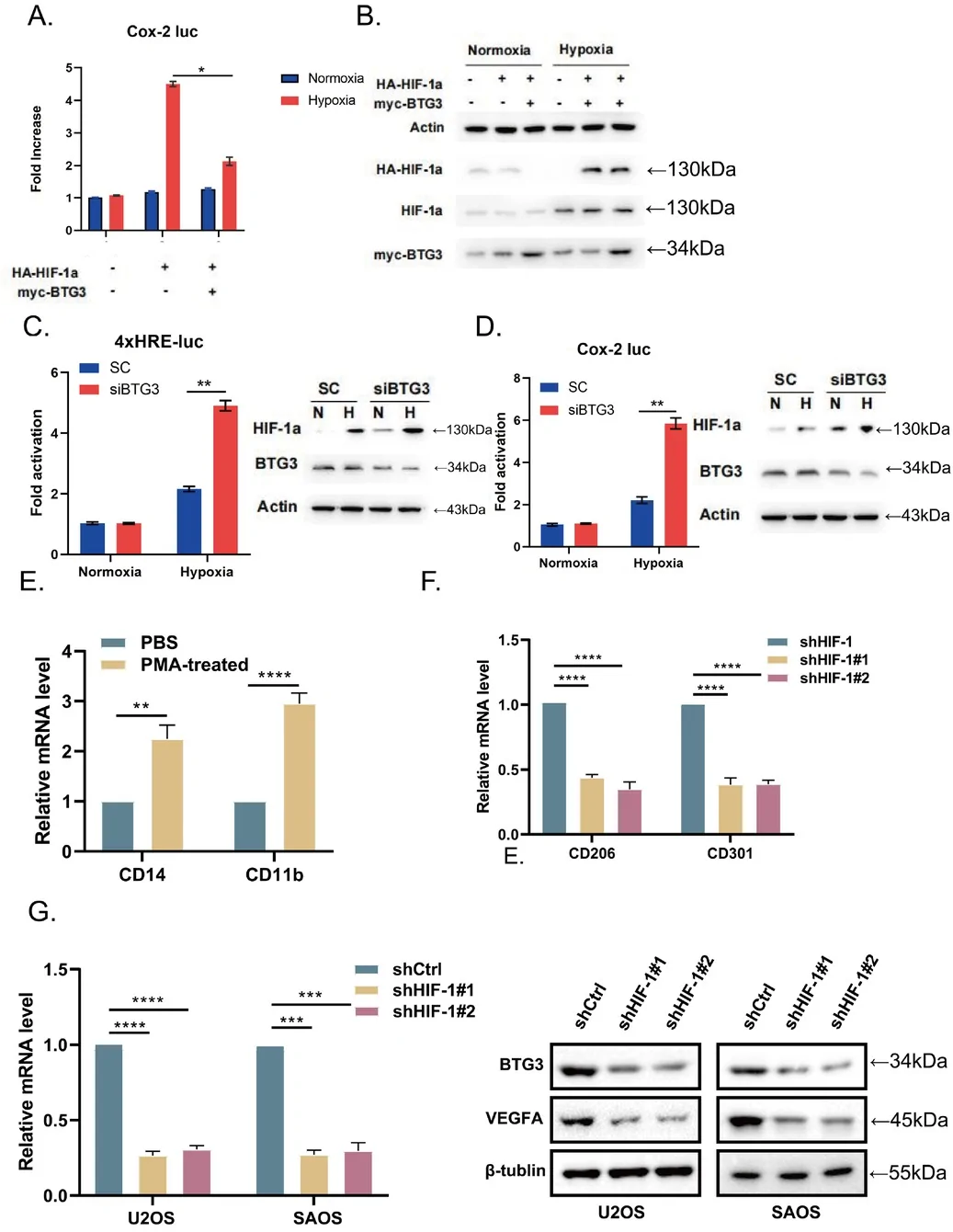
BTG3 negatively regulates HIF-1α transcriptional activity and suppresses COX-2 expression. (A) Cox-2 promoter-driven luciferase activity assay following transfection of HA-HIF-1α and myc-BTG3 under normoxia and hypoxia conditions. Data represent the mean ± SD from three independent experiments. (B) WB detection of HA-HIF-1α, myc-BTG3, and actin under normoxic and hypoxic conditions corresponding to panel A. (C) Left: Luciferase activity (fold activation) after transfecting cells with 4xHRE-luc reporter plasmid followed by treatment with SC or siBTG3. Right: WB analysis of HIF-1α, BTG3, and actin in SC and siBTG3-treated groups under normoxic (N) and hypoxic (H) conditions. (D) Left panel: Cox-2-luc reporter plasmid-transfected cells treated with SC and siBTG3, followed by luciferase activity assay. Right panel: WB analysis of HIF-1α, BTG3, and actin in SC and siBTG3-treated groups under normoxic (N) and hypoxic (H) conditions. (E) qRT-PCR analysis confirming THP-1 cell differentiation into macrophage-like cells (increased CD14 and CD11b) after PMA treatment (*n* = 3). (F) Knockdown of HIF-1 inhibited M2 macrophage polarization (*n* = 3). (G) (Left) qPCR showed that HIF-1 knockdown reduced VEGFA mRNA expression (*n* = 3). (Right) WB confirmed that HIF-1 knockdown significantly decreased VEGFA protein levels in OS cells (*n* = 3). ^*^P < 0.05, ^**^P < 0.01, ^***^P < 0.001, ^****^P < 0.0001. OS: osteosarcoma; qRT-PCR: quantitative real-time reverse transcription polymerase chain reaction; HIF-1α: hypoxia-inducible factor-1α; BTG3: B cell translocation gene 3; siBTG3: small interfering B cell translocation gene 3; HA: hemagglutinin; myc: myelocytomatosis oncogen; SD: standard deviation; 4xHRE-luc: 4 copies of hypoxia response element-luciferas; THP-1: Hospital pediatrics-1; SC: control siRNA; WB, western blot; VEGFA: vascular endothelial growth factor A; mRNA: messenger ribonucleic acid.

To determine whether BTG3 regulates HIF-1α through direct interaction, we performed Co-IP assays. Exogenous myc-BTG3 co-precipitated with HA-HIF-1α, and endogenous BTG3 also interacted with HIF-1α in U2OS cells (Supplementary Figure S5). These data indicate that BTG3 physically binds to HIF-1α to suppress its transcriptional activity.

To further validate the inhibitory role of endogenous BTG3 on HIF-1α transcriptional activity, we knocked down BTG3 using specific siRNA (siBTG3) and assessed its

effect on HRE-driven luciferase activity. As shown in Figure 6C (left panel), knockdown of BTG3 significantly enhanced 4xHRE-luciferase activity compared to the scrambled control (SC), indicating that loss of BTG3 releases HIF-1α from transcriptional repression. The corresponding WB analysis (Figure 6C, right panel) revealed that siBTG3 treatment markedly increased HIF-1α protein levels under both normoxic (N) and hypoxic (H) conditions, while BTG3 knockdown efficiency was confirmed by reduced BTG3 protein signals.

Similarly, we examined the effect of BTG3 knockdown on COX-2 promoter activity. As shown in Figure 6D (left panel), silencing BTG3 significantly enhanced Cox-2-luciferase activity compared to the SC control. WB analysis (Figure 6D, right panel) again confirmed that HIF-1α protein levels were elevated upon BTG3 knockdown under both normoxic and hypoxic conditions, consistent with the luciferase reporter results.

Taken together, these results demonstrate that BTG3 functions as a negative regulator of HIF-1α: it suppresses HIF-1α transcriptional activity and protein accumulation. Loss of BTG3 leads to HIF-1α hyperactivation, which in turn drives the expression of downstream targets such as COX-2, providing a mechanistic basis for the protumorigenic effects of BTG3 deficiency in OS.

### HIF-1α activation driven by BTG3 deficiency promotes macrophage M2 polarization and angiogenesis

To investigate the role of macrophages in the OS microenvironment, we first induced THP-1 monocytes to differentiate into M2-type macrophages through *in vitro* experiments. THP-1 cells were seeded at a density of 1×10^6^ cells/mL in 6-well plates and stimulated with 100 ng/mL phorbol 12-myristate 13-phorbatate (PMA) for 24 hours to induce differentiation into unpolarized macrophages (M0 type). Fresh complete medium was then added, supplemented with 20 ng/mL IL-4 and 10 ng/mL IL-13, and cells were cultured for an additional 48 hours to induce M0-type macrophage polarization toward the M2 type. Concurrently, interferon-γ (IFN-γ) was added for 48 hours. We assessed expression levels of M1 and M2 macrophage markers CD86 and CD206 via flow cytometry and IF staining. Results demonstrated that induced cells highly expressed CD86 and CD206, respectively, confirming successful M2 macrophage induction (Supplementary Figure S6).

We next explored the functional consequences of BTG3/HIF-1α axis activation on the TME. Using PMA-differentiated THP-1 macrophages (Figure 6E), we found that conditioned medium from BTG3-knockdown OS cells promoted macrophage polarization towards an M2 phenotype (increased CD206), an effect that was blunted by concurrent HIF-1α knockdown (Figure 6F). Furthermore, HIF-1α knockdown in OS cells led to a significant decrease in the production of the key pro-angiogenic factor vascular endothelial growth factor A (VEGF-A), at both mRNA and protein levels (Figure 6G).

Functional assays confirmed the critical role of this axis in angiogenesis. Conditioned medium from HIF-1α-knockdown OS cells significantly inhibited the ability of human umbilical vein endothelial cells (HUVECs) to form tube-like structures (Figure 7A, B), migrate through transwell membranes (Figure 7C), and close a scratch wound (Figure 7D). To further validate this mechanism in the tumor context, we performed parallel experiments using OS cells. Consistent with the findings in hFOB cells, BTG3 knockdown in U2OS/SAOS cells significantly promoted HUVEC tube formation and migration, and these pro-angiogenic effects were similarly resistant to PX-478 inhibition (Figure 7E, F; Supplementary Figure S7A, B). Notably, the pro-angiogenic phenotype was more pronounced in the OS cell context compared to the normal osteoblastic cells, suggesting that BTG3 deficiency in tumor cells creates a particularly favorable microenvironment for angiogenesis. Collectively, these results indicate that BTG3 loss can bypass HIF-1α inhibition to drive angiogenesis, positioning BTG3 as a key upstream regulator of the HIF-1α pathway in OS.

**Figure 7:**
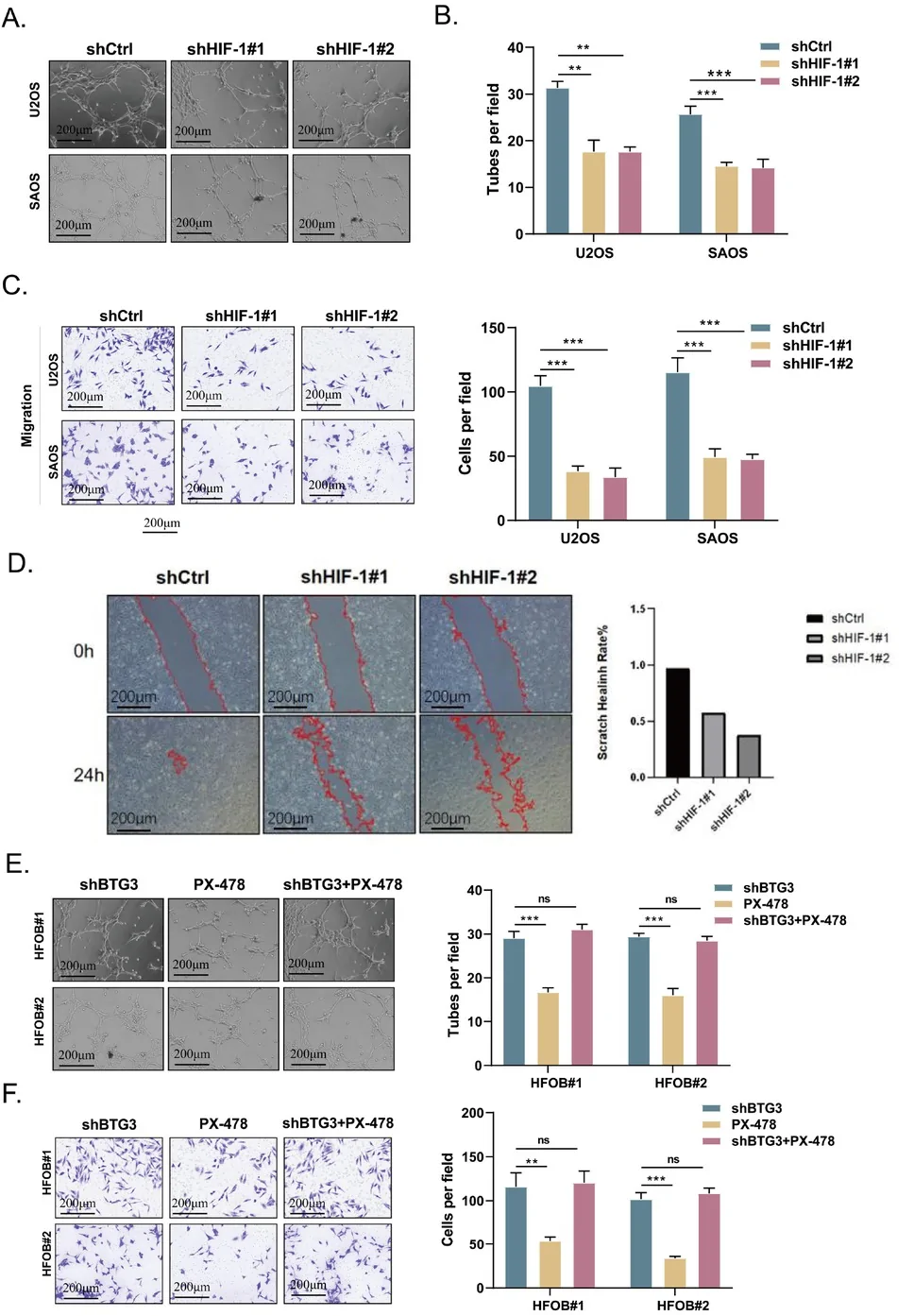
Functional roles of the HIF-1 signaling pathway and BTG3 deficiency in angiogenesis and endothelial cell migration. (A, B) Tube formation assay using HUVECs cultured with conditioned media. (Top) Representative images of tube structures. (Bottom) Quantitative analysis of tube formation (*n* = 3). Knockdown of HIF-1 in OS cells significantly inhibited angiogenesis. (C) Transwell migration assay of HUVECs. (Top) Representative images of migrated cells. (Bottom) Quantitative analysis (*n* = 3). Conditioned media from HIF-1 knockdown OS cells significantly reduced HUVEC migration. (D) Wound healing assay of HUVECs. (Top) Representative images at 0 h and 24 h. (Bottom) Quantitative analysis of scratch closure rate (*n* = 3). Conditioned media from HIF-1 knockdown cells impaired HUVEC migration. *P* < 0.05. (E) Tube formation assay using conditioned media from normal osteoblastic hFOB cells. BTG3 knockdown promoted HUVEC tube formation, and this effect was resistant to HIF-1 inhibitor PX-478 (*n* = 3). (F) Transwell migration assay with hFOB-conditioned media showing BTG3 knockdown promoted HUVEC migration, partially resistant to PX-478 (*n* = 3). ^*^P < 0.05, ^**^P < 0.01, ^***^P < 0.001, ^****^P < 0.0001. HIF-1: hypoxia-inducible factor 1; HUVECs: human umbilical vein endothelial cells; BTG3: B cell translocation gene 3; shBTG3: short hairpin B cell translocation gene 3; U2OS: U-2 osteosarcoma; SAOS: sarcoma osteogenics; Ctrl: control; hFOB: human fetal osteoblast.

## Discussion

In this study, we employed an integrated approach combining bioinformatics analysis, clinical specimen validation, *in vitro* functional assays, and *in vivo* animal models to elucidate a novel signaling axis in OS: TRIM65-BTG3-HIF-1α. This axis plays a central driving role in OS malignant progression by regulating two key processes within the TME: M2 macrophage polarization and angiogenesis. Our findings provide a theoretical framework for understanding the immunosuppressive microenvironment of OS and identify molecular components that may serve as candidates for future therapeutic development, pending functional validation in clinically relevant models.

The primary finding of this study was the confirmation of the predominant infiltration of M2-type TAMs in the OS microenvironment and its strong correlation with poor patient prognosis (Figure 1), which is consistent with observations in many solid tumors.^[43,44]^ Furthermore, by constructing an M2 MRG signature, we focused on a key molecule: BTG3. As a known candidate tumor suppressor gene, BTG3 plays important roles in various cancers.^[38,39]^ However, we discovered a perplexing phenomenon in OS: BTG3 was significantly highly transcribed at the mRNA level, but its protein expression was markedly downregulated (Figure 4, S1). This paradoxical “up-transcription-down-translation” phenomenon suggested the existence of a unique post-transcriptional regulatory mechanism for BTG3 in OS.

Subsequently, we successfully uncovered the core of this mechanism. Through proteomic screening and functional validation, we identified that the E3 ubiquitin ligase TRIM65 is highly expressed in OS and acts as a “molecular switch”, directly binding to the BTG3 protein and mediating its ubiquitination and degradation (Figure 5, S2). This discovery perfectly explains the low protein expression of BTG3. Functionally, TRIM65, by degrading BTG3, significantly promoted the proliferation, migration, and invasion capabilities of OS cells (Figure 5) and tumorigenicity *in vivo* (Supplementary Figure S4), thereby positioning TRIM65 as a novel oncogenic factor in OS.

The most critical breakthrough of this study lies in elucidating the downstream effects of BTG3 loss and its remodeling of the TME. We confirmed that BTG3 is a key negative regulator of the HIF-1α signaling pathway(Figure 6). Under normal physiological conditions, BTG3 may suppress HIF-1α transcriptional activity through a certain mechanism. In OS, TRIM65-mediated degradation of BTG3 releases the “brake” on HIF-1α, leading to sustained activation of its signaling pathway. The activated HIF-1α signaling then drives OS malignant progression in two ways: (1) Inducing an immunosuppressive microenvironment. HIF-1α activation directly promotes macrophage polarization towards the M2 phenotype (Figure 6F), creating an immune-privileged microenvironment for tumor growth. (2) Promoting angiogenesis. HIF-1α upregulates key factors such as VEGF-A (Figure 6G) and significantly enhances the tube formation, migration, and repair capabilities of endothelial cells, providing “nutrients” for rapid tumor growth and metastasis (Figure 7A-D).

A particularly important finding is that BTG3 deficiency partially antagonized the effects of the HIF-1α inhibitor PX-478 in both normal osteoblastic cells and OS cells (Figure 7E, F; Supplementary Figure S7A, B). Notably, this resistance was more pronounced in the tumor cell context, suggesting that BTG3 loss in OS creates a robust pro-angiogenic phenotype that is difficult to suppress by HIF-1α inhibition alone. This discovery raises the hypothesis that loss of BTG3 function in OS could potentially contribute to resistance against HIF-1α-targeted interventions, though direct evidence from combination therapy experiments is currently lacking. BTG3 may be located upstream of HIF-1α or reside in a parallel, crucial regulatory pathway. When BTG3 is deficient, even if HIF-1α is inhibited, tumor cells might still drive angiogenesis and immunosuppression through alternative pathways. This provides a new perspective for understanding the resistance mechanisms of targeted therapy in OS.

Naturally, this study has several limitations: (1) external validation of the prognostic signature was performed using the GSE21257 dataset (53 samples) as an independent validation cohort. It is important to note that we initially intended to use our institution’s prospectively collected OS samples as an additional external validation cohort. However, the majority of these patients are still under active follow-up, with insufficient maturity of survival data (most patients remain alive or have not reached the defined survival endpoints). Consequently, these samples could not be utilized for survival analysis and prognostic model validation at this time. This represents a limitation of the current study, as true multi-center prospective validation using platform-matched clinical samples remains to be completed. We are continuing to follow our patient cohort and plan to incorporate these data in future studies to further validate the generalizability of the M2 MRS. (2) The mechanistic evidence, while internally consistent, lacks definitive proof. We were unable to complete the gold-standard rescue experiment—expressing a ubiquitination-resistant BTG3 mutant in TRIM65-overexpressing cells—because constitutive stabilization of BTG3 led to cell viability problems, suggesting that tightly regulated BTG3 turnover may be essential for cellular homeostasis. Similarly, *in vivo* combination therapy experiments (TRIM65 knockdown plus HIF-1α inhibition) could not be completed within the timeframe of this study due to the need for extensive protocol optimization. The evidence we present—TRIM65-mediated BTG3 ubiquitination, reciprocal protein changes, and phenotypic alignment between TRIM65 loss and BTG3 knockdown—is suggestive but not conclusive. These experiments remain priorities for follow-up work.

The TRIM65-BTG3 ubiquitination interaction itself was previously reported in renal cell carcinoma.^[38]^ Our study does not claim novelty at this upstream mechanistic level. Rather, we extend this axis by identifying HIF-1α activation as a novel downstream consequence, linking it to M2 macrophage polarization and angiogenesis in the OS context. However, we have not demonstrated what, if anything, is unique about the bone environment in regulating this pathway. Whether bone matrix components, mechanical stress, or interactions with osteoclasts/ osteoblasts specifically modulate TRIM65-BTG3-HIF-1α signaling remains entirely unexplored. This represents a significant gap, as our experiments were conducted under standard tissue culture conditions and subcutaneous tumor settings that do not recapitulate the native bone microenvironment.

Our assertion that HIF-1α activation promotes M2 macrophage polarization also requires careful interpretation. The data show that conditioned medium from HIF-1α-active OS cells enhances M2 markers in THP-1-derived macrophages, but this effect could equally reflect HIF-1α-driven secretion of polarizing factors (IL-10, TGF-β, *etc*.) rather than direct cell-autonomous action. The relative contribution of direct versus indirect mechanisms remains unresolved.

Finally, all *in vivo* experiments utilized subcutaneous xenograft models. While these established the functional importance of the TRIM65-BTG3-HIF-1α axis for tumor growth, they cannot capture the specific biology of the bone microenvironment—interactions with mineralized matrix, mechanical loading, or the resident bone cell compartment—that may critically influence this pathway in patients.

In summary, this study identifies the TRIM65-BTG3-HIF-1α axis as a regulator of OS-microenvironment interactions, with supporting evidence from bioinformatics, clinical specimens, and functional experiments. The findings are biologically plausible and internally consistent, but important gaps remain: Definitive mechanistic proof through rescue experiments, robust external validation in independent cohorts, and bone-specific context validation through orthotopic models. These limitations define the boundaries of the current work and point toward priorities for future investigation.

## Conclusion

In summary, this study reveals the TRIM65-BTG3-HIF-1α signaling axis as a key mechanism promoting the progression of OS. The core molecular events include: (1) upstream regulation. The E3 ubiquitin ligase TRIM65 is highly expressed in OS and degrades the tumor suppressor protein BTG3 *via* the ubiquitin-proteasome pathway; (2) core signaling. Loss of BTG3 relieves the suppression on HIF-1α signaling, leading to hyperactivation of the HIF-1α pathway; (3) downstream effects. The activated HIF-1α signaling axis drives TME reprogramming; on one hand, it induces macrophage polarization towards the M2 phenotype, establishing an immunosuppressive microenvironment; on the other hand, it promotes the expression of factors like VEGF-A, stimulating tumor angiogenesis; (4) hypothesis and future directions. BTG3 deficiency represents a potential mechanism that could contribute to resistance against HIF-1α-targeted interventions. While this signaling axis presents interesting targets for further investigation, our current evidence is insufficient to support immediate clinical application.

Therefore, modulation of TRIM65 activity, investigation of BTG3 restoration, or exploration of HIF-1α signaling inhibition may warrant future preclinical investigation. The development of combination strategies, potentially in conjunction with existing immunotherapies, could be explored in subsequent studies to address the immunosuppressive microenvironment of OS. However, given the preliminary nature of our findings, including the lack of rescue experiments and combination therapy data, clinical translation remains speculative and would require extensive validation in orthotopic models and subsequent clinical trials.

## Supplementary Information

Supplementary materials are only available at the official site of the journal (www.intern-med.com).

## Supplementary Material

Supplementary Material Details
