# Supplementary material for: TRIM65-BTG3-HIF-1α axis drives osteosarcoma progression via M2 macrophage polarization and angiogenesis
Source: J Transl Int Med. 2026 Oct 23;14(5):779–98. doi: 10.1515/jtim-2026-0075 (PMC13726653; doi:10.1515/jtim-2026-0075)
Supplement: Supplementary file 1 [file jtim-2026-0075_sm.pdf]

Supplementary materials

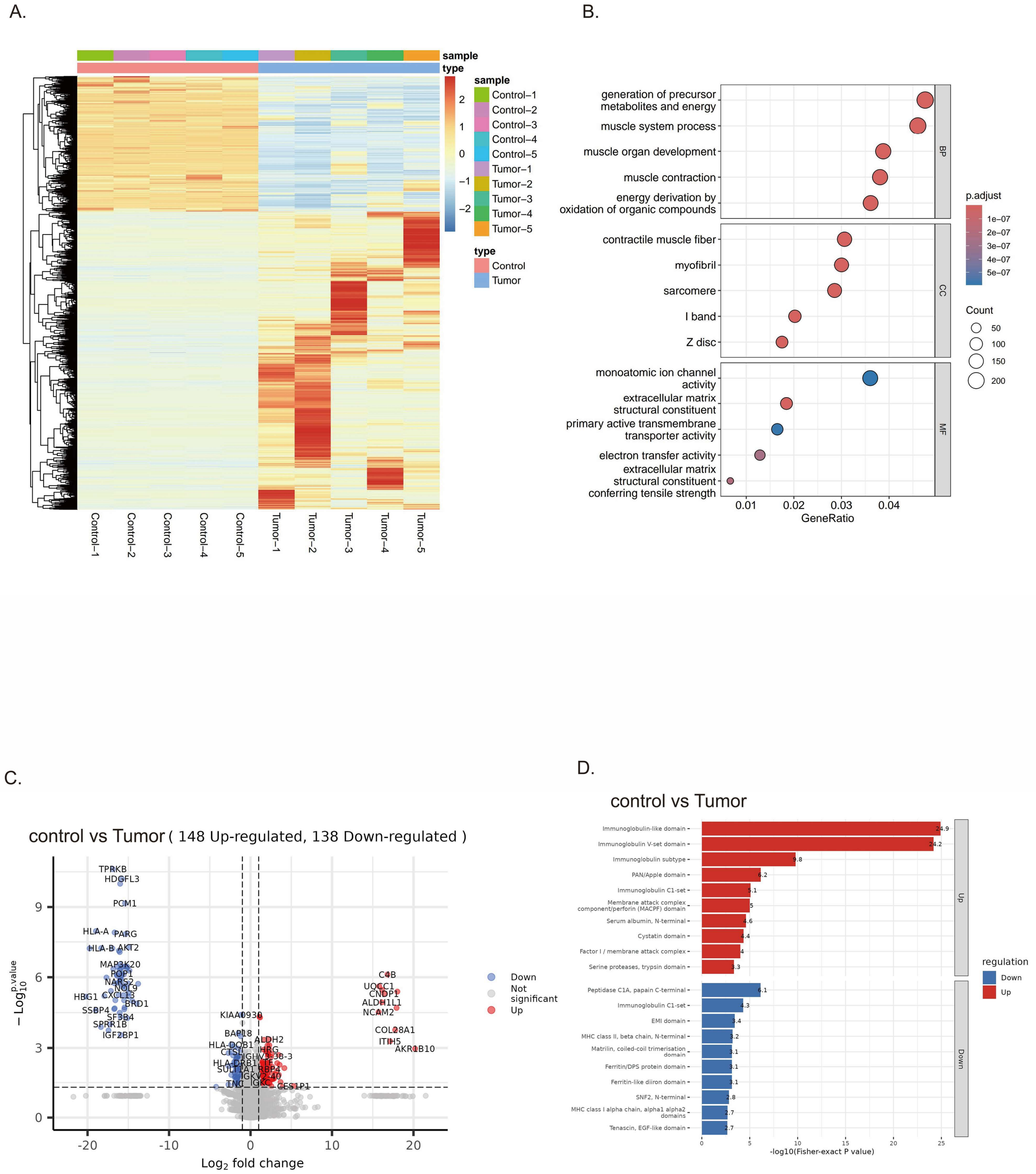

Supplementary Figure S1. Transcriptomic and proteomic profiling of OS reveals the expression paradox of BTG3. (A) Heatmap of differentially expressed mRNAs between OS and adjacent normal tissues from 5 patients. (B) Gene ontology (GO) enrichment analysis of the differentially expressed mRNAs, showing enrichment in biological processes such as muscle contraction and extracellular matrix organization. (C) Volcano plot from DIA quantitative proteomics analysis of 3 paired samples. (D) Protein domain enrichment analysis of the differentially expressed proteins, highlighting domains involved in immune-related functions like immunoglobulin and perforin. OS:

osteosarcoma; BTG3: B cell translocation gene 3; DIA: data-independent acquisition; mRNA: messenger ribonucleic acid; GO: gene ontology.

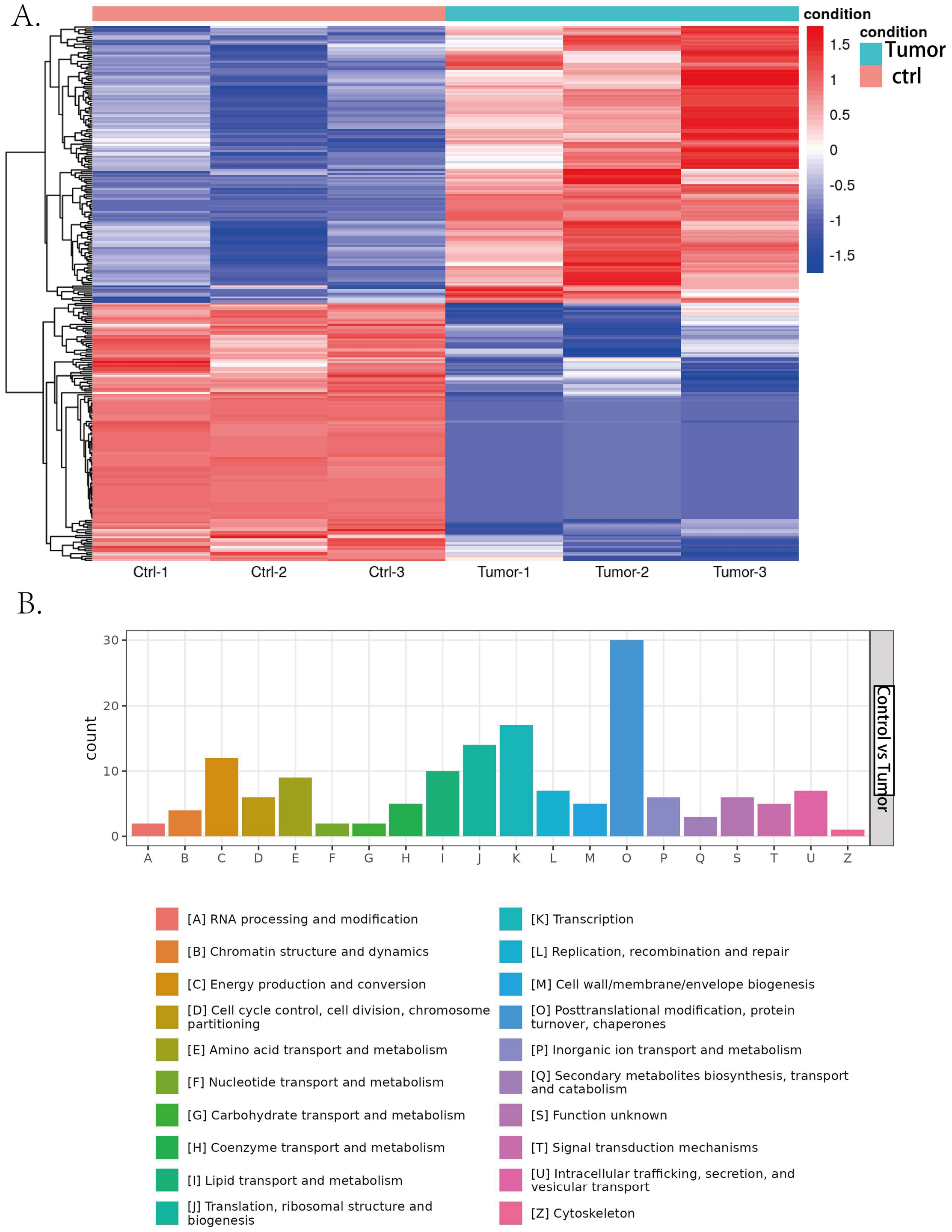

Supplementary Figure S2. Proteomic screening implicates TRIM65 as a potential upstream regulator of BTG3. (A) Heatmap of differentially expressed proteins between OS and adjacent tissues. (B) COG functional classification of the differentially expressed

proteins, indicating enrichment in categories related to post-translational modification, protein turnover, andchaperones. OS: osteosarcoma; COG: clusters of orthologous groups; TRIM65: tripartite motif-containing 65; BTG3: B cell translocation gene 3; Ctrl: control; RNA: ribonucleic acid.

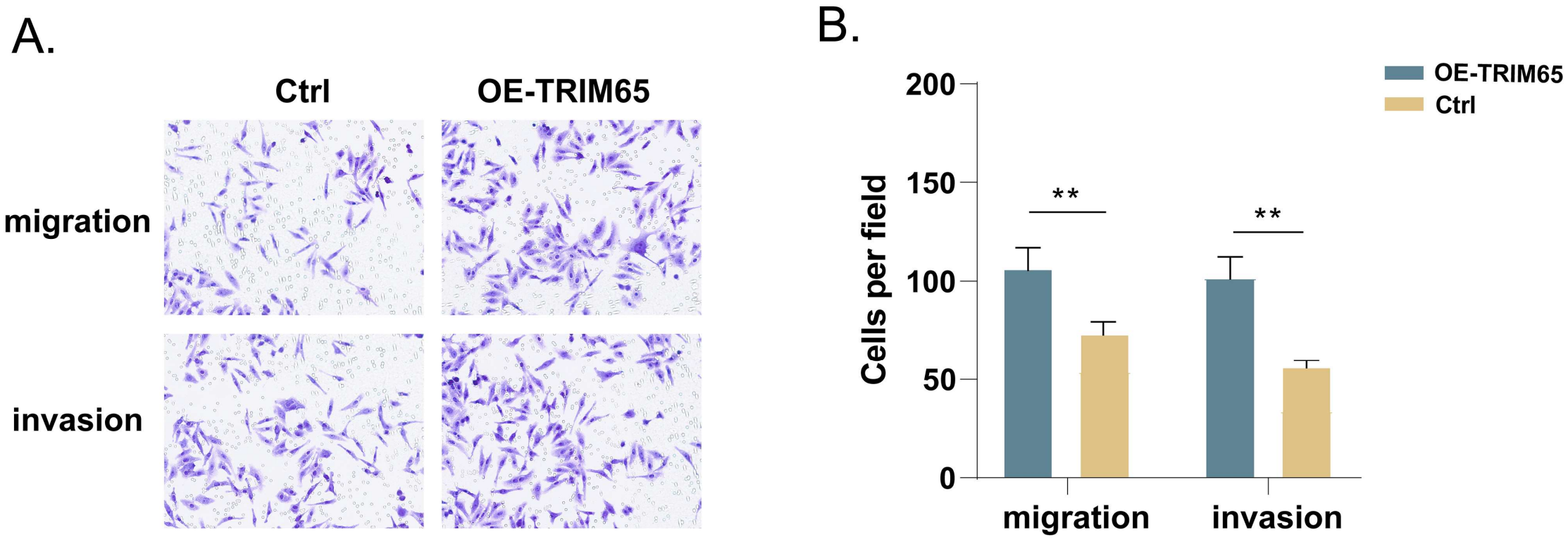

Supplementary Figure S3. TRIM65 overexpression promotes OS cell migration and invasion. (A) Representative images of Transwell migration (upper) and invasion (lower) assays in U2OS cells overexpressing TRIM65 or empty vector. (B) Quantitative analysis of cell numbers per field. Migration (left) and invasion (right) were significantly increased in TRIM65-overexpressing cells. Data are mean ± SD; $P < 0.05$ ($n = 3$). OS: osteosarcoma; U2OS: U-2 osteosarcoma; TRIM65: tripartite motif-containing 65; Ctrl: control; SD: standard deviation.

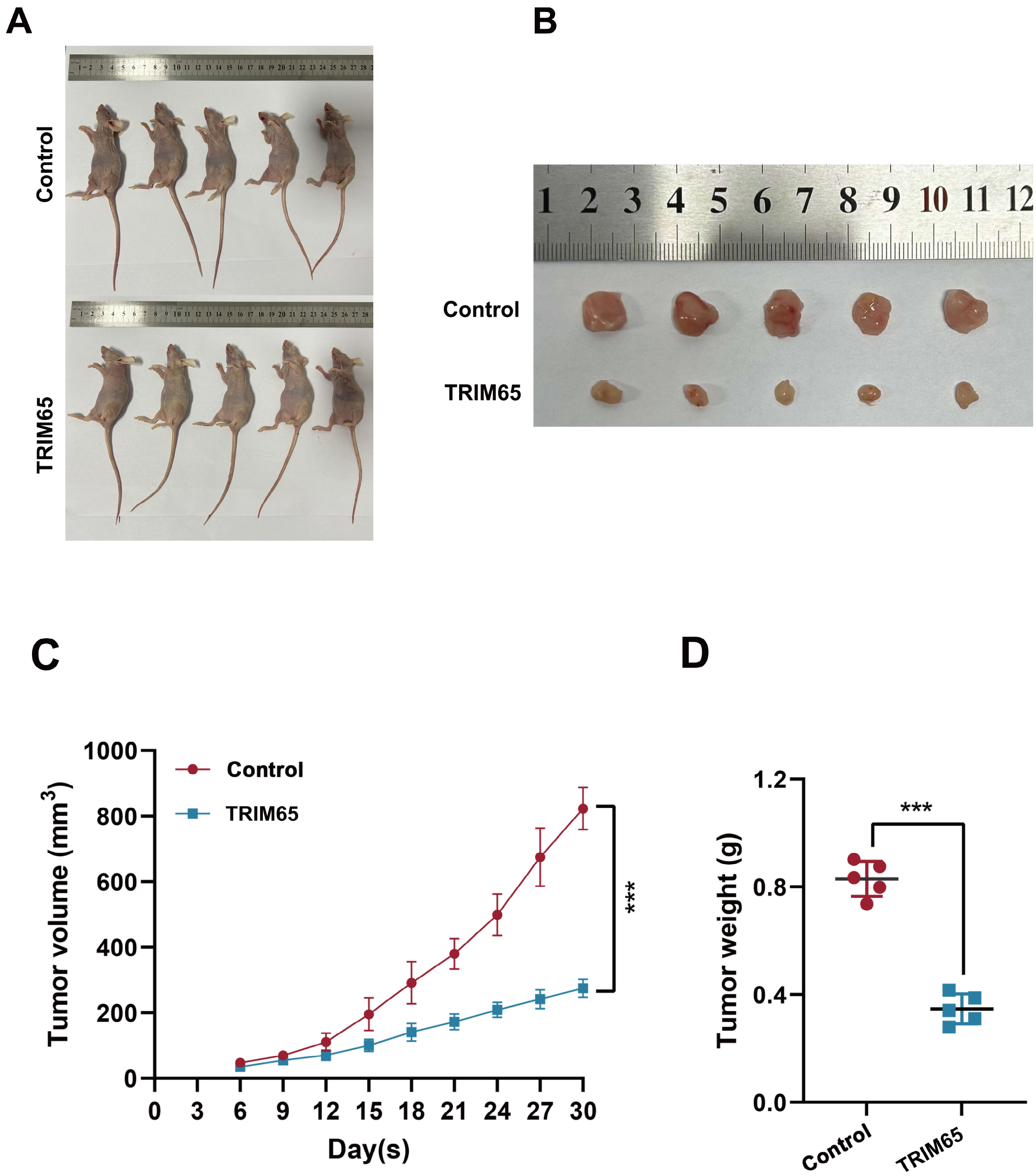

Supplementary Figure S4. *In vivo* validation confirms that TRIM65 knockdown suppresses OS growth. (A) Representative images of mice bearing subcutaneous tumors from the shCtrl and shTRIM65 groups. (B) Representative photographs of tumors resected from nude mice at the experimental endpoint. Tumors from the shTRIM65 group were significantly smaller. (C) Tumor growth curves measured over 28 days. The growth rate of tumors in the shTRIM65 group was significantly slower than in the shCtrl group from day 16 onward ($P < 0.05$). (D) Final tumor weights at the endpoint. Tumors from the shTRIM65 group were significantly lighter. Data are presented as mean ± SD; $P < 0.05$. OS: osteosarcoma; TRIM65: tripartite motif-containing 65; shTRIM65: short hairpin tripartite motif-containing 65; SD: standard deviation.

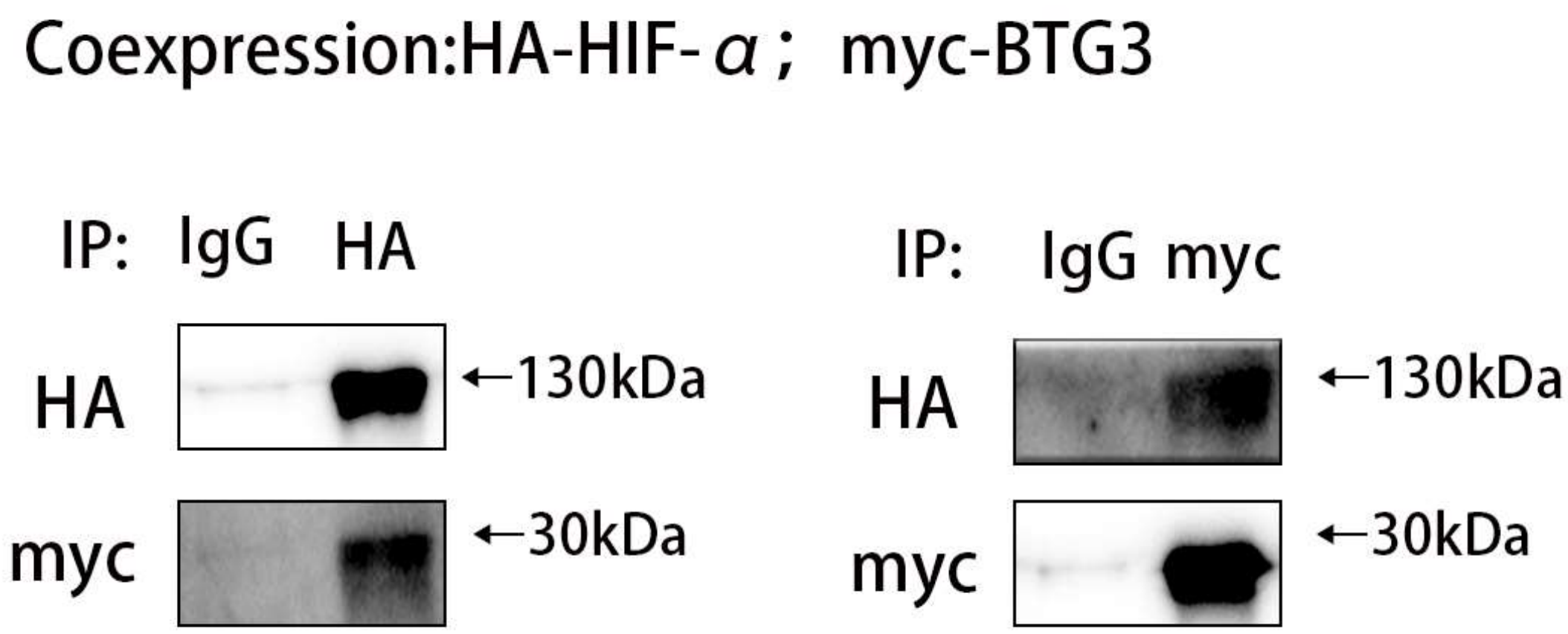

Supplementary Figure S5. BTG3 interacts with HIF-1α in OS cells. OS: osteosarcoma; BTG3: B cell translocation gene 3; HIF-1α: hypoxia-inducible factor 1α; HA: hemagglutinin; myc: myelocytomatosis oncogen.

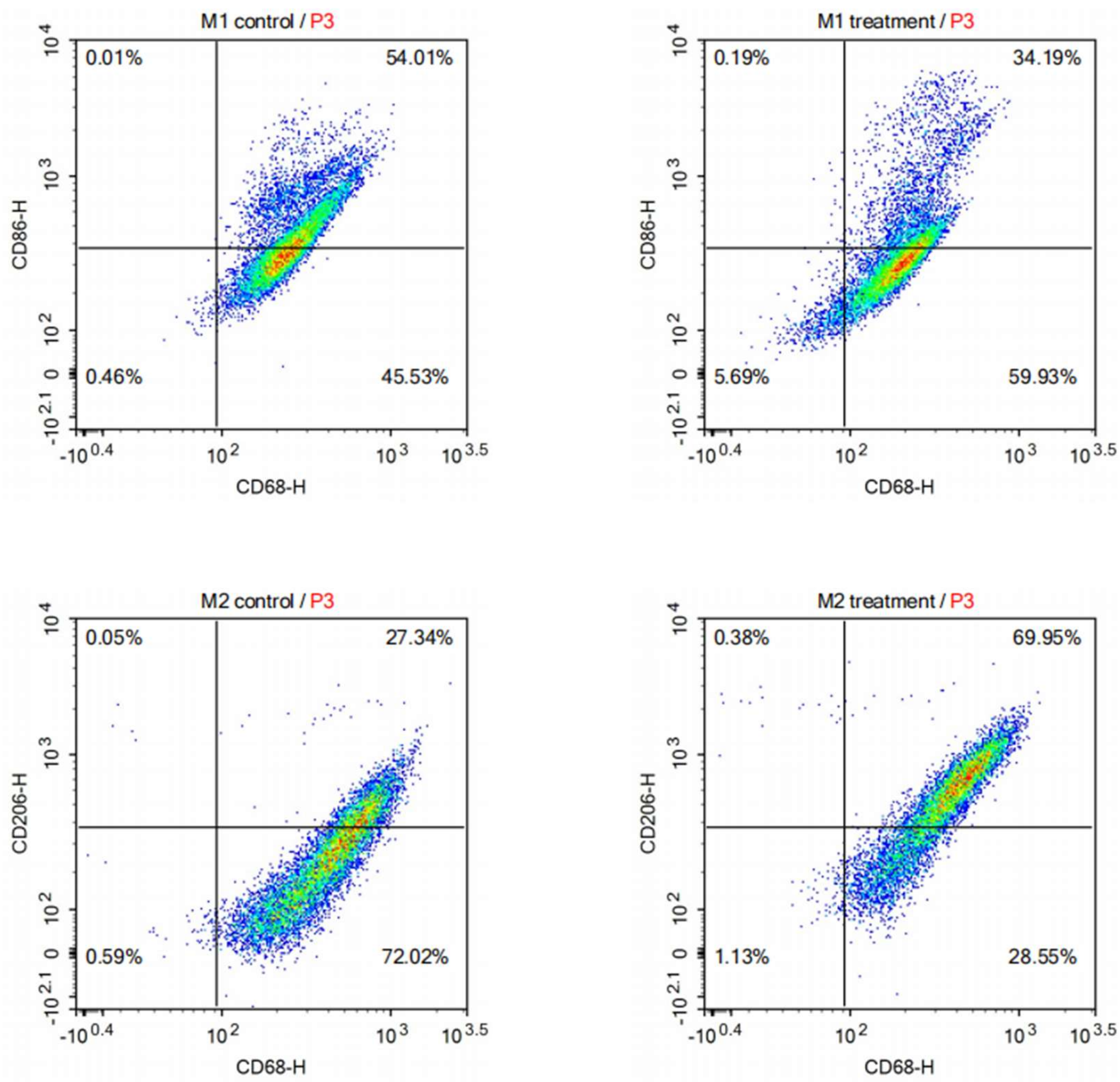

Supplementary Figure S6. Flow cytometry identification of macrophage induced differentiation results showed that macrophages induced by IFN-γ and LPS overexpressed M1 marker CD86; Macrophages induced by IL-4 and IL-13 showed high expression of the M2 marker CD206. IFN-γ: interferon-**γ** ; LPS: lipopolysaccharide; CD: cluster of differentiation; IL: interleukin.

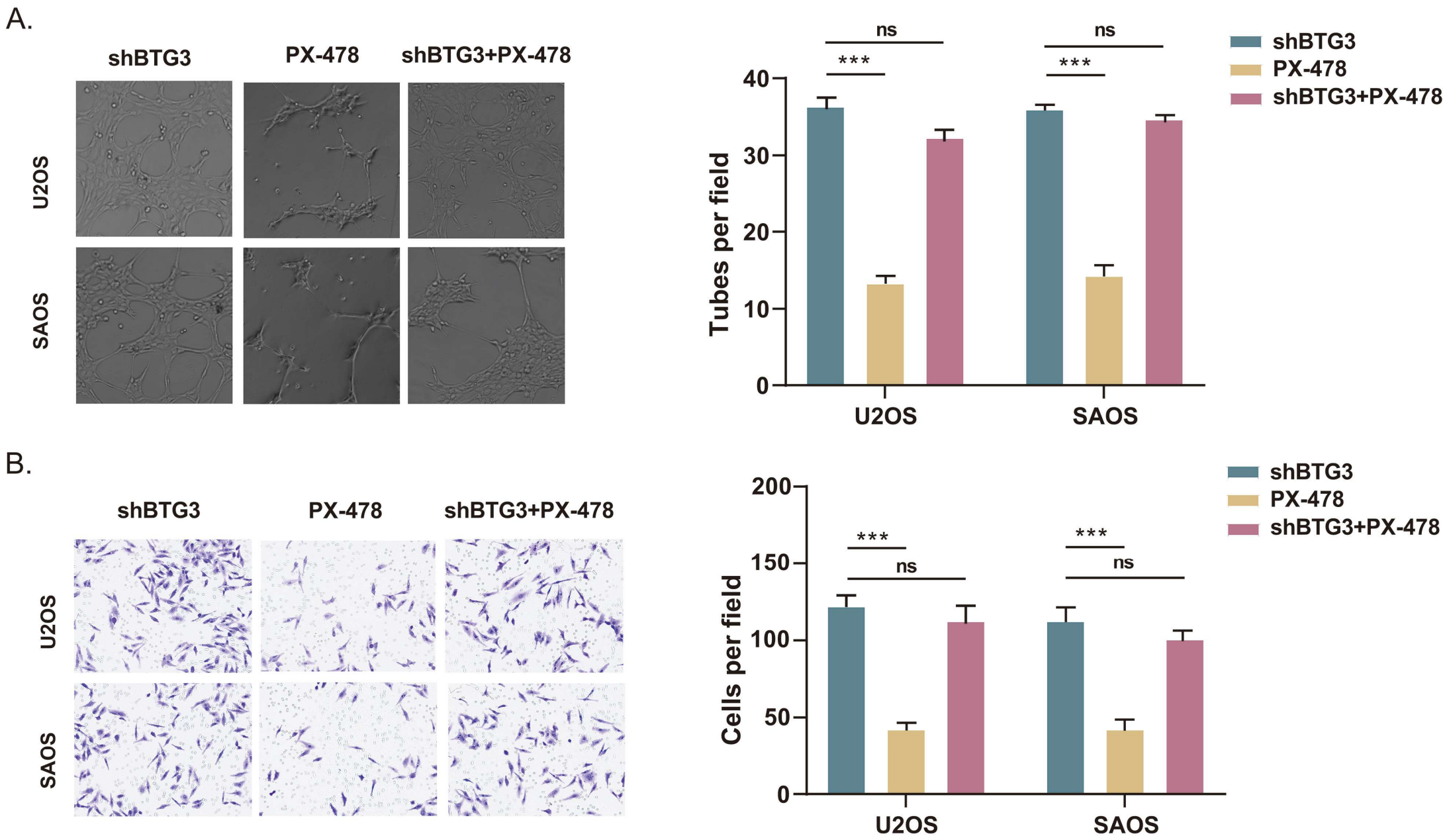

Supplementary Figure S7. BTG3 deficiency in OS cells promotes angiogenesis and resists HIF-1α inhibition. (A) Tube formation assay using conditioned media from OS (U2OS/143B) cells. BTG3 knockdown significantly enhanced tube formation compared to hFOB cells, with resistance to PX-478 inhibition (n = 3, P < 0.05). (B) Transwell migration assay with OS cell-conditioned media confirming the potent pro-migratory effect of BTG3 deficiency and its resistance to HIF-1α inhibition (n = 3, P < 0.05). OS: osteosarcoma; BTG3: B cell translocation gene 3; shBTG3: short hairpin B cell translocation gene 3; HIF-1α: hypoxia-inducible factor 1α; U2OS: U-2 osteosarcoma; SAOS: sarcoma osteogenics; hFOB: human fetal osteoblast.
